# Asymptotic Properties for Cumulative Probability Models for Continuous Outcomes

**DOI:** 10.3390/math11244896

**Published:** 2023-12-07

**Authors:** Chun Li, Yuqi Tian, Donglin Zeng, Bryan E. Shepherd

**Affiliations:** 1 Division of Biostatistics, Department of Population and Public Health Sciences, University of Southern California, Los Angeles, CA 90033, USA; 2 Department of Biostatistics, Vanderbilt University, Nashville, TN 37203, USA; 3 Department of Biostatistics, University of Michigan, Ann Arbor, MI 48109, USA

**Keywords:** cumulative probability model, semiparametric transformation model, uniform consistency, asymptotic distribution, 62G99

## Abstract

Regression models for continuous outcomes frequently require a transformation of the outcome, which is often specified a priori or estimated from a parametric family. Cumulative probability models (CPMs) nonparametrically estimate the transformation by treating the continuous outcome as if it is ordered categorically. They thus represent a flexible analysis approach for continuous outcomes. However, it is difficult to establish asymptotic properties for CPMs due to the potentially unbounded range of the transformation. Here we show asymptotic properties for CPMs when applied to slightly modified data where bounds, one lower and one upper, are chosen and the outcomes outside the bounds are set as two ordinal categories. We prove the uniform consistency of the estimated regression coefficients and of the estimated transformation function between the bounds. We also describe their joint asymptotic distribution, and show that the estimated regression coefficients attain the semiparametric efficiency bound. We show with simulations that results from this approach and those from using the CPM on the original data are very similar when a small fraction of the data are modified. We reanalyze a dataset of HIV-positive patients with CPMs to illustrate and compare the approaches.

## 1. Introduction

Regression analyses of continuous outcomes often require a transformation of the outcome to meet modeling assumptions. In practice, convenient but ad hoc transformations such as a logarithm or square root are often used on right-skewed outcomes; an alternative is to use the Box–Cox family [1] of transformations, which is effectively a family of power functions plus the logarithm transformation. Because the correct transformation for the continuous outcome is often unknown and it may not fall in a prespecified family, it is desirable to estimate the transformation in a flexible way. Semiparametric transformation models have been introduced to address this issue [2,3]. These models involve a latent intermediate variable and two model components: one connecting the latent variable to the outcome variable through an unknown transformation and the other connecting the latent variable to the input variables as in traditional regression models with unknown beta coefficients.

Early parameter estimation for semiparametric transformation models was based on the marginal likelihood of the vector of outcome ranks [2–4]. Although this marginal likelihood can be simplified to the partial likelihood in Cox proportional hazards models [5], it cannot be simplified for other transformation models, and various approximations had to be used. As the marginal likelihood only involves the beta coefficients, additional ad hoc procedures were developed to estimate the transformation [2,3].

Later developments for fitting semiparametric linear transformation models primarily focused on right-censored data, initially relying on estimating equations [6,7]. Zeng and Lin [8] developed nonparametric maximum likelihood estimators (NPMLEs) based on likelihoods for time-to-event data and showed the consistency and asymptotic distribution of their estimators. NPMLEs are desirable because they are fully efficient. For continuous outcomes, more recent developments have used B-splines and Bernstein polynomials to flexibly model the transformation [9,10], but these estimators of the transformation are not fully nonparametric.

With continuous outcomes, one way to nonparametrically estimate the transformation is to treat the outcome as if it is ordinal—without any categorization—and fit to cumulative probability models (CPMs; also called cumulative link models) [11]. Liu et al. [11] showed that the CPM’s multinomial likelihood for continuous outcomes is equivalent to the nonparametric likelihood for semiparametric transformation models. This result led to new NPMLEs for semiparametric transformation models for continuous outcomes using computationally simple ordinal regression methods. They showed with simulations that CPMs perform well in a wide range of scenarios. The method has since been used in applications to analyze various outcomes [12–19].

However, there is no established asymptotic theory for this new NPMLE approach for continuous outcomes. One main hurdle is that the unknown transformation of the continuous outcome variable can have an unbounded range of values, which makes it hard to establish asymptotic properties across the whole range. The approaches that were used to prove asymptotic properties for the NPMLE of the baseline cumulative hazard function for time-to-event outcomes cannot be applied directly to study transformation models for a continuous outcome [8] because the latter has no bounds on its range and no clear definition of a baseline hazard function.

To address this issue, we establish several asymptotic properties in this paper for CPMs when they are applied to continuous outcomes with slight modification. Briefly, a lower bound L and an upper bound U for the outcome are chosen prior to analysis, the outcomes below L are set as the lowest category and those above U as the highest category, and then a CPM is fitted to the modified data. We prove that, in this approach, the nonparametric estimate of the transformation function is consistent (i.e., converges in probability to its true value) uniformly in the interval [L, U]. We then show that the estimator of the beta coefficients and that of the transformation jointly converge to a tight Gaussian process, and that the estimator of the beta coefficients attains the semiparametric efficiency bound. The latter implies that this estimator is (asymptotically) as efficient as possible under the assumptions of the model. We show with simulations and real data that the results from this approach and those from the CPM on the original data are very similar when only a small fraction of data are outside the bounds.

## 2. Method

### 2.1. Cumulative Probability Models

Let Y be the outcome of interest and Z be a vector of p covariates. The semiparametric linear transformation model is

(1) $$Y=H\left(\beta^{T}Z+\epsilon \right),$$

where H is a transformation function assumed to be non-decreasing but unknown otherwise, β is a vector of coefficients, and ϵ is independent of Z and is assumed to follow a continuous distribution with cumulative distribution function G(⋅). An alternative expression of model (1) is

(2) $$A(Y)=\beta^{T}Z+\epsilon ,$$

where $A=H^{-1}$ is the inverse of H. For mathematical clarity, we assume H is left continuous and define A(y)=sup{z:H(z)≤y}; then, A is non-decreasing and right-continuous.

Model (1) is equivalent to the cumulative probability model (CPM) presented in Liu et al. [11]:

(3) $$G^{-1}\left\{\text{P}\left(Y\leq y|Z\right)\right\}=A\left(y\right)-\beta^{T}Z,\text{ for any }y,$$

where $G^{-1}(\cdot )$ serves as a link function. One example of the distribution for ϵ is the standard normal distribution. In this case, the CPM becomes a normal linear model after a transformation, which includes log-linear models and linear models with a Box–Cox transformation as special cases. The CPM becomes a Cox proportional hazards model when ϵ follows the extreme value distribution, i.e. $G(x)=1-\text{exp}\left(-e^{x}\right)$, or a proportional odds model when ϵ follows the logistic distribution, i.e. $G(x)=e^{x}/\left(1+e^{x}\right)$.

Suppose the data are i.i.d. and denoted as $\left(Y_{i},Z_{i}\right),i=1,\ldots ,n$. Liu et al. [11] proposed to model the transformation A nonparametrically. The corresponding nonparametric (NP) likelihood is

$$\prod_{i=1}^{n}\left[G\left\{A(Y_{i})-\beta^{T}Z_{i}\right\}-G\left\{A(Y_{i}^{-})-\beta^{T}Z_{i}\right\}\right],$$

where $A\left(y^{-}\right)={\text{lim}}_{t↑y}\;A(t)$. Since A can be any non-decreasing function, this likelihood will be maximized when the increments of A(⋅) are concentrated at the observed $Y_{i}$; if some increments of A(⋅) are not at the observed $Y_{i}$, its corresponding probability mass at non-observed values can always be reallocated to some observed values to increase the likelihood. Thus, we can maximize this likelihood by only considering step functions A(⋅) that have a jump at every observed $Y_{i}$. This leads to an expression of the likelihood that is the same as the likelihood of the CPM when the outcome variable is treated as if it were ordered categorically with the observed distinct values as the ordered categories. As a result, nonparametric maximum likelihood estimates (NPMLEs) can be obtained by fitting an ordinal regression model to the continuous outcome. Liu et al. [11] showed in simulations that CPMs perform well under a wide range of scenarios. However, it is difficult to prove the asymptotic properties for this approach. Since some $Y_{i}$ can be extremely large or small and the observations at the tails are often sparse, there is high variability in the estimate of A at the tails. Moreover, the unboundedness of the transformation at the tails makes it difficult to control the compactness of the estimator of A, thus making most of asymptotic theory no longer applicable. In this paper, we prove asymptotic properties for CPMs when they are applied to continuous outcomes with slight modification. We describe the modification in Section 2.2 and show the asymptotic results in Section 2.3.

### 2.2. Cumulative Probability Models on Modified Data

In view of the challenges above, we hereby describe an approach in which the outcomes are modified at the two ends before a CPM is fit. We will then describe the asymptotic properties of this approach in Section 2.3 and show with simulations that the results from this approach and those of the CPM on the original data are similar when a small fraction of data are modified.

More specifically, we predetermine a lower bound L and an upper bound U, and consider all observations with $Y_{i}\leq \;L$ as a single ordered category, which we conveniently denote as L, and those with $Y_{i}\geq U$ as a single ordered category, denoted as U. The bounds L and U should satisfy $\text{P}\left(L<Y<U\right)>0,\text{ P}(Y\leq L)>0$, and P(Y≥U)>0. The new outcome variable, denoted as $Y_{i}^{\prime }$, follows a mixture distribution. When $Y_{i}^{\prime }\in (L,U)$, the distribution is continuous with the same cumulative distribution function as that for $Y_{i}$; that is, $\text{P}\left(Y_{i}^{\prime }\leq y|Z_{i}\right)=\text{P}\left(Y_{i}\leq y|Z_{i}\right)=G\left\{A(y)-\beta^{T}Z_{i}\right\}$ for y∈(L,U). When $Y_{i}^{\prime }=L$ or $Y_{i}^{\prime }=U$, the distribution is discrete, with $\text{P}\left(Y_{i}^{\prime }=L|Z_{i}\right)=G\left\{A(L)-\beta^{T}Z_{i}\right\}$ and $\text{P}\left(Y_{i}^{\prime }=U|Z_{i}\right)=1-G\left\{A\left(U^{-}\right)-\beta^{T}Z_{i}\right\}$. Then, the nonparametric likelihood for the modified data is

(4) $$\prod_{i=1}^{n}\left({\left[G\left\{A\left(Y_{i}\right)-\beta^{T}Z_{i}\right\}-G\left\{A\left(Y_{i}^{-}\right)-\beta^{T}Z_{i}\right\}\right]}^{I\left(Y_{i}\in \left(L,U\right)\right)}\times G{\left\{A\left(L\right)-\beta^{T}Z_{i}\right\}}^{I\left(Y_{i}\leq L\right)}\times {\left[1-G\left\{A\left(U^{-}\right)-\beta^{T}Z_{i}\right\}\right]}^{I\left(Y_{i}\geq U\right)}\right),$$

where I(S) is the indicator function for event S with value 1 if S occurs and 0 otherwise.

Since A(⋅) can be any non-decreasing function over the interval [L,U), the likelihood (4) will be maximized when the increments of A(⋅) are concentrated at the observed $Y_{i}^{\prime }$. Hence, it suffices to consider only step functions with a jump at each distinct value of $Y_{i}^{\prime }\in [L,U]$.

### 2.3. Asymptotic Results

From now on, we assume the outcome is continuous. Without loss of generality, we assume that in our models (1)–(3), the support of Y contains 0, the vector Z contains an intercept and has p dimensions, and A(0)=0. Furthermore, the two bounds satisfy L<0, U>0,P(L<Y<U)>0,P(Y≤L)>0, and P(Y≥U)>0. To establish the asymptotic properties described below, we further assume that

1.  G(x) is thrice-continuously differentiable, $G^{\prime }(x)>0$ for any $x,\;G^{\prime \prime }(x)\text{sign}(x)<0$ for |x|≥M, where M>0 is a constant, and
$$\underset{x\to \infty }{\text{lim inf}}\;G^{\prime }\left(x\right)/\left\{1-G\left(x\right)\right\}>0,\underset{x\to -\infty }{\text{ lim inf}}\;G^{\prime }\left(x\right)/G\left(x\right)>0.$$
2. The covariance matrix of Z is non-singular. In addition, Z and β are bounded so that $\beta^{T}Z\in [-m,m]$ almost certainly for some large constant m.
3.  A(y) is continuously differentiable in (−∞,∞).

Condition 1 imposes restrictions on G(x) at both tails; it holds for many residual distributions, including the standard normal distribution, the extreme value distribution and the logistic distribution. Conditions 2 and 3 are minimal assumptions for establishing asymptotic properties for linear transformation models.

Let $\left(\hat{\beta },\hat{A}\right)$ denote the nonparametric maximum likelihood estimate of (β,A) that maximizes the likelihood (4) on the modified data. Then, $\hat{A}$ is a step function with a jump at each of the distinct $Y_{i}^{\prime }$ in the modified data. To establish the asymptotic properties of $\left(\hat{\beta },\hat{A}\right)$, we consider $\hat{A}$ as a function over the closed interval [L,U] by defining $\hat{A}(U)=\hat{A}\left(U^{-}\right)$. We have the following consistency theorem.

**Theorem 1.** *Under conditions 1–3, with probability one,*

$$\underset{y\in \left[L,U\right]}{\text{sup}}\;\left|\hat{A}\left(y\right)-A\left(y\right)\right|+∥\hat{\beta }-\beta ∥\to 0.$$

The proof of Theorem 1 is in Appendix A. Core steps of the proof include showing that $\hat{A}$ is bounded in [L,U] with probability one. Then, since $\hat{A}(\cdot )$ is bounded and increasing in [L,U], via the Helly selection theorem [20], for any subsequence, there exists a further subsequence that converges to a non-decreasing, right-continuous function at its continuity points. Thus, without confusion, we assume that $\hat{A}\to A^{*}$ weakly in [L,U] and $\hat{\beta }\to \beta^{*}$. We then show that with probability one, $A^{*}(y)=A(y)$ for y∈[L,U] and $\beta^{*}=\beta$. With this result, the consistency is established. Furthermore, since A is continuously differentiable, we conclude that $\hat{A}(y)$ converges to A(y) uniformly in [L,U] with probability one.

We next describe the asymptotic distribution of $\left(\hat{\beta },\hat{A}\right)$. The asymptotic distribution of $\hat{A}$ will be expressed as that of a random functional in a metric space. We first define some notation. Let BV[L,U] be the set of all functions defined over [L,U] for which the total variation is at most one. Let lin⁡(BV[L,U]) be the set of all linear functionals over BV[L,U]; that is, every element f in lin⁡(BV[L,U]) is a linear function f:BV[L,U]→R. For any f∈lin⁡(BV[L,U]), its norm is defined as $∥f∥={\text{sup}}_{h\in BV[L,U]}\;f[h]$. A metric over lin⁡(BV[L,U]) can then be derived subsequently. Given any non-decreasing function A over [L,U], a corresponding linear functional in lin⁡(BV[L,U]), also denoted as A, can be defined such that for any h∈BV[L,U],

$$A[h]=\int_{L}^{U}h(x)dA(x).$$

Similarly, for an nonparametric maximum likelihood estimate $\hat{A}$, its corresponding linear functional in lin⁡(BV[L,U]) is $\hat{A}$, such that for any h∈BV[L,U],

$$\hat{A}\left[h\right]=\int_{L}^{U}h\left(x\right)d\hat{A}\left(x\right).$$

The functional $\hat{A}$ is a random element in the metric space lin⁡(BV[L,U]). For any y∈(L,U), there exists an h∈BV[L,U], such that $\hat{A}(y)=\hat{A}[h]$. For example, suppose the estimated jump sizes at the distinct outcome values of a dataset, $\left\{a_{1},\ldots ,a_{J}\right\}$, are $\left\{{\overset{ˆ}{s}}_{1},\ldots ,{\overset{ˆ}{s}}_{J}\right\}$. Then, at $y_{0}>0,\hat{A}\left(y_{0}\right)=\sum_{0<a_{j}\leq y_{0}}\;{\overset{ˆ}{s}}_{j}=\hat{A}\left[h_{0}\right]$, where $h_{0}(y)=I\left(0<y\leq y_{0}\right)$; and, similarly, at $y_{0}<0,\hat{A}\left(y_{0}\right)=-\sum_{y_{0}<a_{j}<0}\;{\overset{ˆ}{s}}_{j}=\hat{A}\left[h_{0}\right]$, where $h_{0}(y)=I\left(y_{0}<y<0\right)$.

**Theorem 2.** *Under conditions 1–3,* $n^{1/2}(\hat{\beta }-\beta ,\hat{A}-A)$ *converges weakly to a tight Gaussian process in* $R^{p}\times lin(BV[L,U])$. *Furthermore, the asymptotic variance of* $n^{1/2}(\hat{\beta }-\beta )$ *attains the semiparametric efficiency bound*.

The proof of Theorem 2 is in Appendix B and makes use of weak convergence theory for empirical processes and semiparametric efficiency theory. Its proof relies on verifying all the technical conditions in the Master Z-Theorem in [21]. In particular, it entails verification of the invertibility of the information operator for (β,A).

Because the information operator for (β,A) is invertible, the arguments given in [22] imply that the asymptotic variance-covariance matrix of $\left(\hat{\beta },\hat{A}\left[h_{1}\right],\ldots ,\hat{A}\left[h_{m}\right]\right)$ for any $h_{1},\ldots ,h_{m}\in BV[L,U]$ can be consistently estimated based on the information matrix for $\hat{\beta }$ and the jump sizes of $\hat{A}$. Specifically, suppose the estimated jump sizes at the distinct outcome values of a dataset, $\left\{a_{1},\ldots ,a_{J}\right\}$, are $\left\{{\overset{ˆ}{s}}_{1},\ldots ,{\overset{ˆ}{s}}_{J}\right\}$. Let ${\hat{I}}_{n}$ be the estimated information matrix for both $\hat{\beta }$ and $\left\{{\overset{ˆ}{s}}_{1},\ldots ,{\overset{ˆ}{s}}_{I}\right\}$. Then, the variance-covariance matrix for $\left(\hat{\beta },\hat{A}\left[h_{1}\right],\ldots ,\hat{A}\left[h_{m}\right]\right)$ is estimated as $V^{T}{\hat{I}}_{n}^{-1}V$, where

$$V=\left(\begin{array}{cc} I_{p\times p} & 0\\ 0 & H \end{array}\right)$$

and H is a J×m matrix with elements ${\left\{h_{k}\left(a_{j}\right)\right\}}_{1\leq j\leq J,1\leq k\leq m}$.

## 3. Simulation Study

### 3.1. Simulation Set-Up

CPMs have been extensively simulated elsewhere to justify their use and have been largely seen to have good behavior [11]. Here we perform a more limited set of simulations to illustrate three major points which are particularly relevant to our study. First, the estimation of A(y) using CPMs can be biased at extreme values of y. Even though $\hat{A}(y)$ may have point-wise consistency for any $y,\hat{A}(y)$ may not be uniformly consistent over all y∈(−∞,∞). Second, in the modified approach, $\hat{A}(y)$ is uniformly consistent over y∈[L,U]. Third, except for the estimation of extreme quantiles and A(y) at extreme levels, the results are largely similar between CPMs fit to the original data and the modified data.

We roughly followed the simulation settings of Liu et al. [11]. Let $X_{1}\sim \text{Bernoulli}(0.5)$, $X_{2}\sim N(0,1)$, and $Y=\text{exp}\left(\beta_{1}X_{1}+\beta_{2}X_{2}+\epsilon \right)$, where $\beta_{1}=1,\beta_{2}=-0.5$, and ϵ~N(0,1). In this set-up, the correct transformation function is A(y)=log⁡(y). We generated datasets $\left\{\left(X_{1},X_{2},Y\right)\right\}$ with sample sizes n=100,1000, and 5000. We fit CPMs that have the correctly specified link function (probit) and model form (linear). The performance of misspecified models was extensively studied via simulations [11]. In CPMs, the transformation A and the parameters $\left(\beta_{1},\beta_{2}\right)$ are semi-parametrically estimated. We evaluated how well the transformation was estimated by comparing $\hat{A}(y)$ with the correct transformation, A(y)=log⁡(y), for various values of y.

We fit CPMs to the original data and CPMs to the modified data with (L,U) set to $\left(e^{-4},e^{4}\right),\left(e^{-2},e^{2}\right)$, and $\left(e^{-1/2},e^{1/2}\right)$; these values correspond to approximately 0.2%, 13%, and 71% of Y being modified, respectively. All simulations had 1000 replications.

### 3.2. Simulation Results

**Figure 1.**
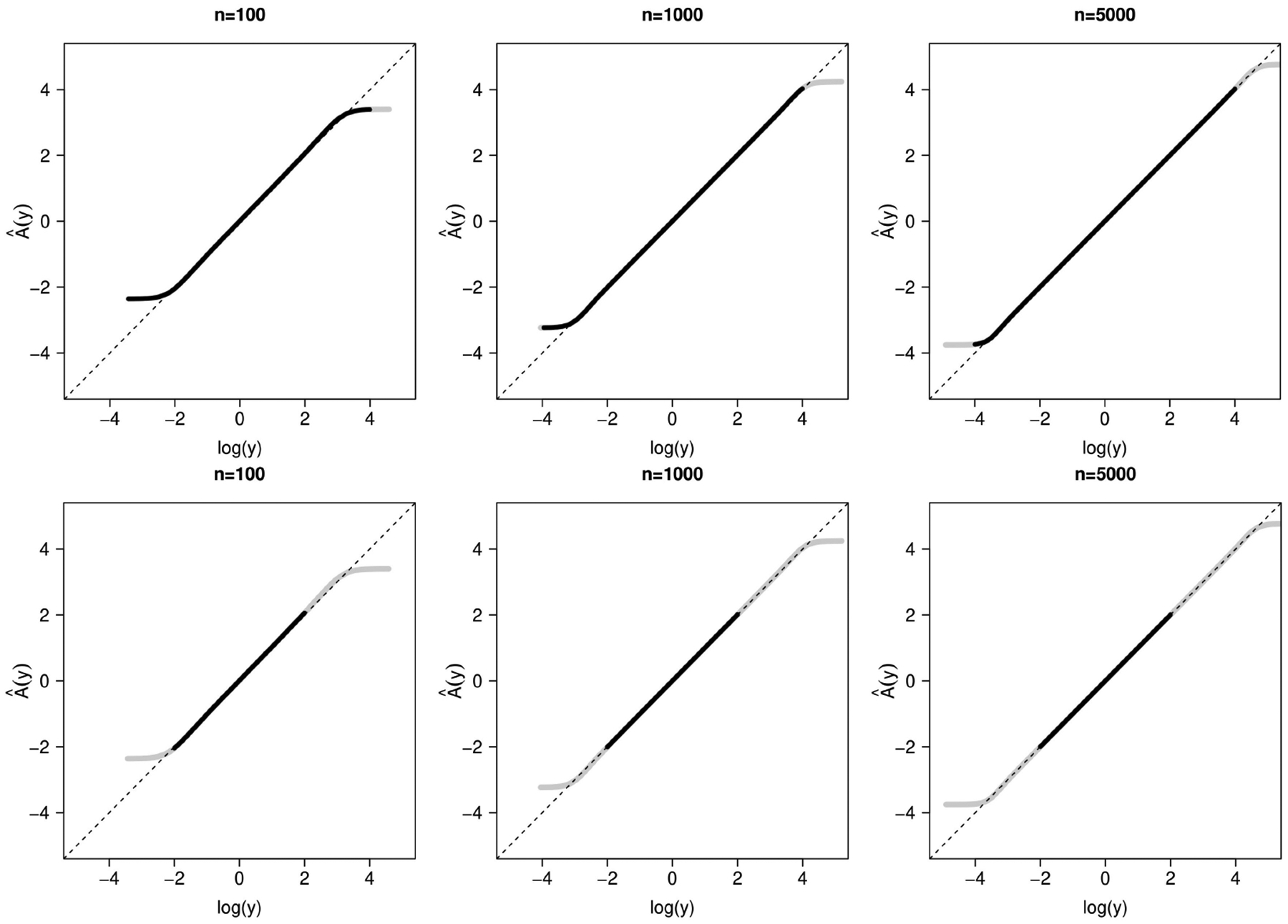

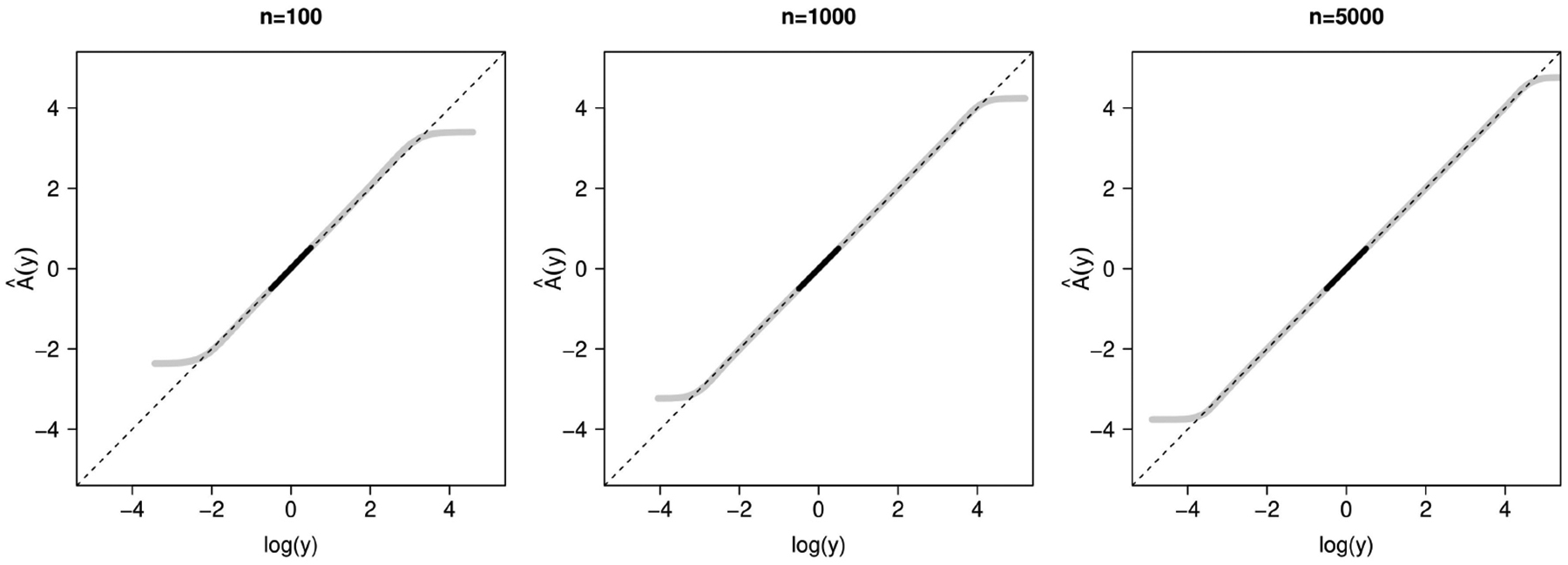
Average estimate of A(y) after fitting properly specified CPMs compared with the true transformation, log⁡(y). Gray curve: original data; black curve: modified data. Dashed lines are the diagonal. Top row: $(L,U)=\left(e^{-4},e^{4}\right)$; middle row: $(L,U)=\left(e^{-2},e^{2}\right)$; bottom row: $(L,U)=\left(e^{-1/2},e^{1/2}\right)$. Left to right: n=100,1000,5000. Based on 1000 replications.

Figure 1 shows the average estimate of A(y) across 1000 simulation replicates compared with the true transformation, log⁡(y). The left, center, and right panels are results based on sample sizes of 100, 1000, and 5000, respectively. With the original data, for all sample sizes, estimates are unbiased when y is around the center of its distribution (i.e., where the bulk of the probability mass lies), approximately in the range $\left[e^{-2},e^{3}\right]$ when n=100, in $\left[e^{-3},e^{4}\right]$ when n=1000, and in a wider range when n=5000. However, at extreme values of y, we see biased estimation. This illustrates that, for a fixed y, one can find a sample size large enough that the estimation of A(y) is unbiased, but that there will always be a more extreme value of y for which $\hat{A}(y)$ may be biased. This motivates the need to categorize values outside a predetermined range (L,U) to achieve the uniform consistency of $\hat{A}(y)$ for y∈[L,U].

**Figure 2.**
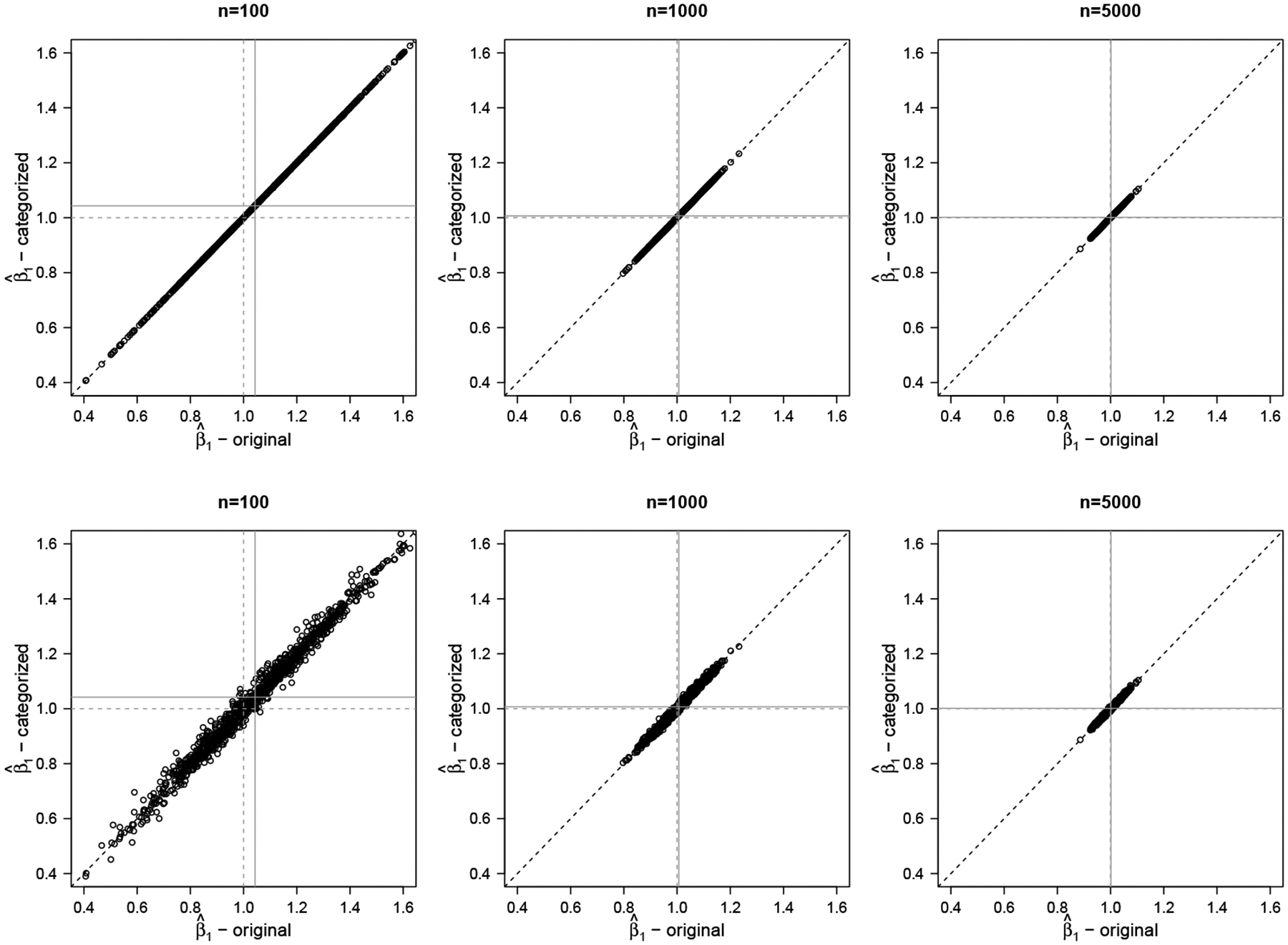

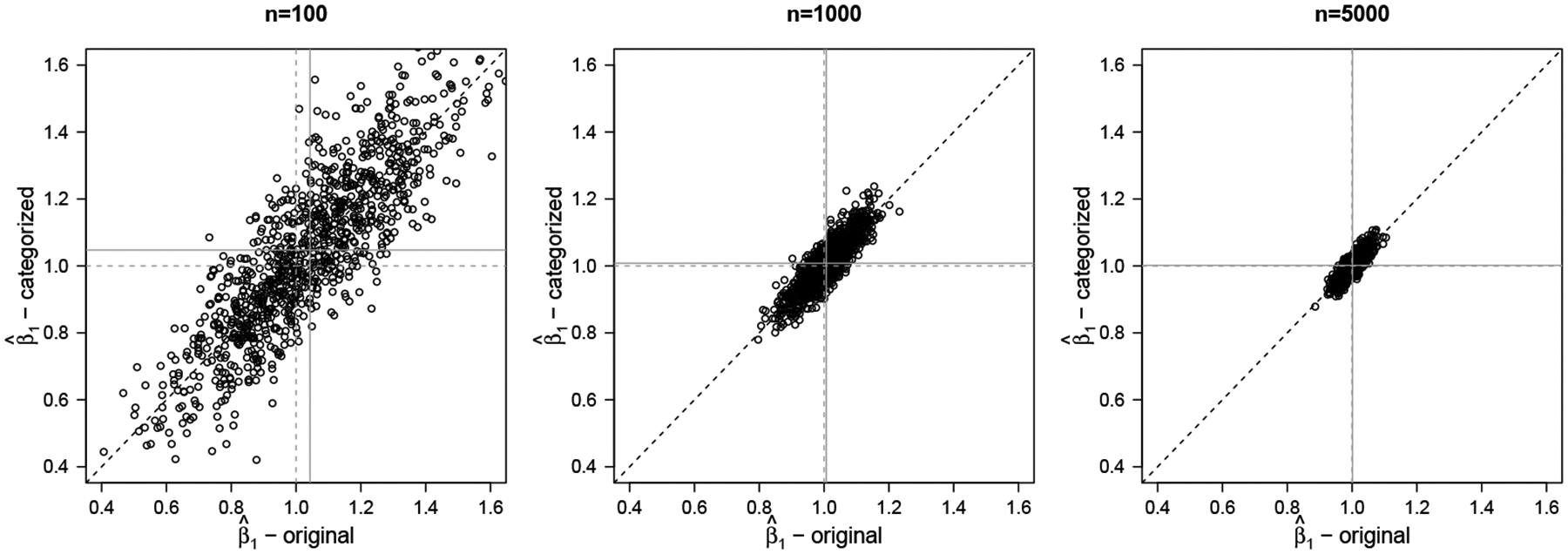
Estimates of $\beta_{1}$ using data categorized outside (L,U) compared with those using the original data and to the truth, $\beta_{1}=1$. Gray lines are mean estimates and dashed gray lines are the truth. Top row: $(L,U)=\left(e^{-4},e^{4}\right)$; middle row: $(L,U)=\left(e^{-2},e^{2}\right)$; bottom row: $(L,U)=\left(e^{-1/2},e^{1/2}\right)$. Left to right: n=100,1000,5000. Based on 1000 replications.

Figure 2 compares estimates of $\beta_{1}$ for the various sample sizes using the original data and using the modified data. As the sample size becomes larger, ${\overset{ˆ}{\beta }}_{1}$ becomes less biased in all approaches. At $n=5000,{\overset{ˆ}{\beta }}_{1}$ is approximately unbiased even with a large proportion of the data having been categorized. Not surprisingly, with increasing proportions of categorized data, ${\overset{ˆ}{\beta }}_{1}$ becomes slightly more variable (Table 1) and slightly less correlated with that estimated from the original data. The results for $\beta_{2}$ have similar patterns (Supplementary Material Figure S1).

Table 1 shows further results for five estimands: $\beta_{1},\beta_{2},A\left(e^{0.5}\right)$, and the conditional median and mean of Y given $X_{1}=0$ and $X_{2}=0$. For each estimand, we compute the bias of the corresponding estimate, its standard deviation across replicates, the mean of estimated standard errors, and the mean squared error. For the estimands $\beta_{1},\beta_{2}$, and $A\left(e^{0.5}\right)$, estimation using the original data appears to be consistent, and the behavior of our estimators with the modified data is as predicted by the asymptotic theory. When n=100, there appears to be only a modest amount of bias, even with 71% categorized; when n=1000 (Table 1) and 5000 (shown in Supplementary Material Table S1), the bias is quite small. Although in Figure 1 we saw that estimates of A(y) for extreme values of y were biased, we see no evidence that this impacts the estimation of $\beta_{1}$ and $\beta_{2}$. The average standard errors are very similar to the empirical results (i.e., the standard deviation of parameter estimates across replicates), suggesting that we are correctly estimating standard errors. These results hold regardless of the proportion categorized in our simulations. With increasing proportions being categorized, as expected, both absolute bias and standard deviation increase, and, as a result, the mean squared error increases. However, all these measures become smaller as the sample size increases.

We cannot compute the standard error for the conditional median. Categorization also prohibits the sound estimation of the conditional mean; one could instead estimate the trimmed conditional mean, e.g., $E\left(Y|X_{1}=0,X_{2}=0,L\leq Y\leq U\right)$, which may substantially differ from $E\left(Y|X_{1}=0,X_{2}=0\right)$. The bias of $\hat{A}(y)$ for extreme values of y had little impact on the estimation of $E\left(Y|X_{1}=0,X_{2}=0\right)$, which is computed using $\hat{A}(y)$ over the entire range of observed y.

## 4. Example Data Analysis

**Figure 3.**
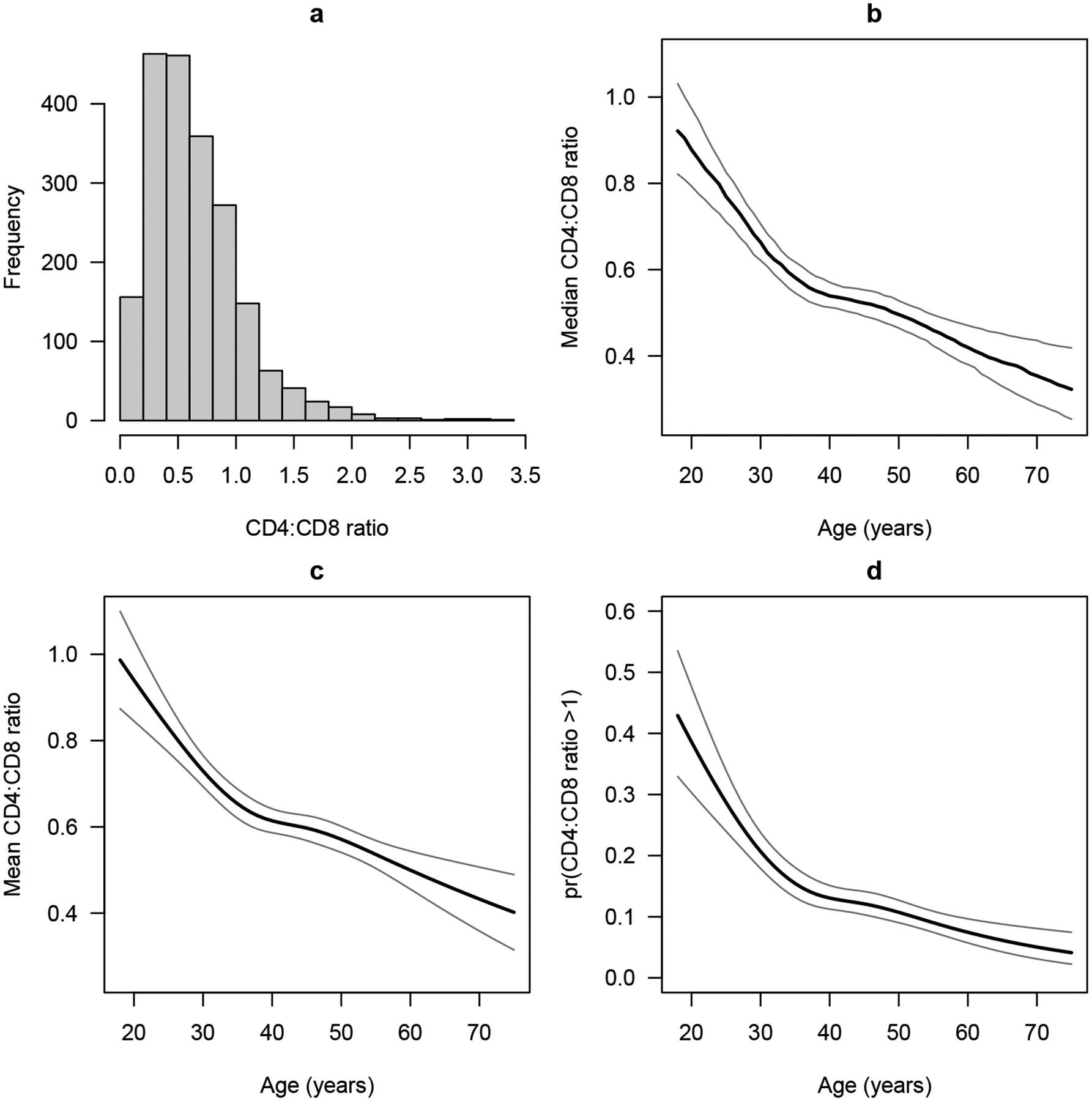
(**a**) Histogram of CD4:CD8 ratio in our dataset. (**b-d**) Estimated outcome measures and 95% confidence intervals as functions of age, holding other covariates constant at their medians/modes. (**b**) Median CD4:CD8 ratio; (**c**) mean CD4:CD8 ratio; (**d**) probability that CD4:CD8 > 1.

CD4:CD8 ratio is a biomarker for measuring the strength of the immune system. A normal CD4:CD8 ratio is between 1 and 4, while people with HIV tend to have much lower values, and a low CD4:CD8 ratio is highly predictive of poor outcomes including non-communicable diseases and mortality. When people with HIV are put on antiretroviral therapy, their CD4:CD8 ratio tends to increase, albeit often slowly and quite variably. Castilho et al. [23] studied factors associated with the CD4:CD8 ratio among 2024 people with HIV who started antiretroviral therapy and maintained viral suppression for at least 12 months. They considered various factors including age, sex, race, the probable route of transmission, hepatitis C co-infection, hepatitis B co-infection, and the year of antiretroviral therapy initiation. Here we re-analyze their data using CPMs. We will focus on the associations of the CD4:CD8 ratio with age and sex, treating the other factors as covariates. The CD4:CD8 ratio tends to be right-skewed (Figure 3a), but there is no standard transformation for analyzing it. In various studies, it has been left untransformed [23], log-transformed [24], dichotomized (CD4:CD8 > 1 vs. ≤ 1) [25], put into ordered categories roughly based on quantiles [26], square-root transformed [27], and fifth-root transformed [28]. In contrast, CPMs do not require the specification of the transformation.

We fit three CPMs: Model 1 using the original data, Model 2 categorizing all CD4:CD8 ratios below L=0.1 and above U=2.0, and Model 3 categorizing below L=0.2 and above U=1.5. In a similar group of patients in a prior study [29], these values of L and U were approximately the 1.5th and 99.5th percentiles, respectively, for Model 2, and the 7th and 95th percentiles for Model 3. In our dataset, there were 19 (0.9%) CD4:CD8 ratios below 0.1 and 21 (1%) above 2.0, and 156 (7.7%) below 0.2 and 74 (3.7%) above 1.5. In our models, age was modeled using restricted cubic splines [30] with four knots at the 0.05, 0.35, 0.65, and 0.95 quantiles. All models were fit using a logit link function; quantile–quantile plots of probability-scale residuals [11] from the models suggested a good model fit (Supplementary Materials Figure S2).

All three models produced nearly identical results. Female sex had regression coefficients 0.6002, 0.6000, and 0.5994 in Models 1, 2, and 3, respectively (likelihood ratio p<0.0001 in all models), suggesting that the odds of having a higher CD4:CD8 ratio, after controlling for all other variables in the model, were about $e^{0.6}=1.82$ times higher for females than for males (95% Wald confidence interval 1.44–2.31). The median CD4:CD8 ratio holding all other covariates fixed at their medians/modes was estimated to be 0.67 (0.60–0.74) for females compared with 0.53 (0.51–0.56) for males; all models had the same estimates to two decimal places. The mean CD4:CD8 ratio holding all other covariates constant was estimated to be 0.73 (0.67–0.79) for females and 0.61 (0.58–0.63) for males from Model 1. The mean estimates from Models 2 and 3 were slightly different (e.g., 0.72 for females); however, the mean should not be reported after categorization because the estimates arbitrarily assigned the categorized values to be L and U.

Older age was strongly associated with a lower CD4:CD8 ratio (p<0.0001 in all models), and the association was non-linear (p=0.0080, 0.0081, 0.0086, respectively). Figure 3b–d show the estimated median and mean CD4:CD8 ratio and the probability that CD4:CD8 > 1 as functions of age, all derived from the CPMs and holding other covariates fixed at their medians/modes. The median CD4:CD8 ratio and P(CD4:CD8 > 1) were not discernibly different between the three models. The mean as a function of age is only shown as derived from Model 1.

## 5. Discussion

We have now established the asymptotic properties for CPMs applied to data categorized at the tails. CPMs are flexible semiparametric regression models for continuous outcomes because the outcome transformation is nonparametrically estimated. We proved uniform consistency of the estimated coefficients $\hat{\beta }$ and the estimated transformation function $\hat{A}$ over the interval [L,U], and showed that their joint asymptotic distribution is a tight Gaussian process. We demonstrated that these estimators perform well with simulations and illustrated their use in practice with a real data example.

Establishing uniform consistency requires a bounded range of the transformation function A, which is achieved by categorizing the outcome variable at both ends. Even if an outcome variable has a bounded support, the transformed values may not be bounded, and categorization will still be needed to establish uniform consistency. The proof of uniform consistency for $\hat{\beta }$ also requires a bounded range of A even though β and A are separate components of the model.

Although the asymptotic properties for a similar nonparametric maximum likelihood approach in survival analysis have been established [8], the proofs here for CPMs with continuous data are different because we consider the nonparametric maximum likelihood estimate for the transformation in CPMs rather than the cumulative hazards function as in survival analysis. In addition, the transformation is estimated in the proofs directionally and separately for the two tails, which also differs from prior work.

For data without natural lower and upper bounds, the choice of L and U might be challenging in practice. In our CD4:CD8 ratio analysis, we were able to select values of L and U that corresponded with small and large CD4:CD8 percentiles in a prior study, therefore likely ensuring that a small fraction of the data would be modified in our analysis. In general, it is desirable to choose bounds so that only a small fraction of the data are categorized, although it should be reiterated that these bounds should be chosen prior to analysis. Both our simulations and our data example suggest that the results are robust to the specific choices of L and U as long as they do not lead to a high proportion of the data being categorized. For example, in our simulations, the results were nearly identical when categorization varied between 0.2% and 13%; in the data example, results were also nearly identical when categorization varied between 1.9% and 11.4%. Therefore, if one chooses to specify L and U, we suggest to select them so that approximately 5% or fewer of the observations would be modified at each end.

Our simulations and data example actually also suggest that without categorization, the estimators also perform well, which may support the use of CPMs with the original data in practice. CPMs applied to the original data do not require specifying L and U, and they permit the calculation of conditional means. However, its asymptotic theory has not been established; hence, there might be some risk to analyses using CPMs on the original data.

Continuous data that are skewed or subject to detection limits are common in applied research. Because of their ability to non-parametrically estimate a proper transformation, their robust rank-based nature, and their desirable properties proven and illustrated in this manuscript, CPMs are often an excellent choice for analyzing these types of data. Extensions of CPMs to more complicated settings, e.g., clustered and longitudinal data, multivariate outcome data, or data with multiple detection limits, are warranted and are areas of ongoing research.

## Supplementary Material

Supplement

## Figures and Tables

**Table 1.** Simulation results for estimates from CPMs on original data and on data categorized outside (L,U);n=100,1000; based on 1000 replications.

n	Estimand		Original Data	Data Categorized Outside (L, U)		
				$\left(e^{-4},e^{4}\right)$	$\left(e^{-2},e^{2}\right)$	$\left(e^{-1/2},e^{1/2}\right)$
100	$\beta_{1}$	bias	0.043	0.043	0.042	0.048
		SD	0.228	0.228	0.229	0.260
		mean SE	0.217	0.217	0.219	0.251
		MSE	0.054	0.054	0.054	0.070
	$\beta_{2}$	bias	−0.022	−0.021	−0.020	−0.022
		SD	0.119	0.119	0.120	0.143
		mean SE	0.110	0.110	0.111	0.133
		MSE	0.015	0.015	0.015	0.021
	$A\left(e^{0.5}\right)$	bias	0.019	0.019	0.019	0.020
		SD	0.177	0.177	0.177	0.183
		mean SE	0.174	0.174	0.175	0.182
		MSE	0.032	0.032	0.032	0.034
	$\text{median}\left(Y|X_{1}=0,\;X_{2}=0\right)$	bias	0.022	0.022	0.023	0.021
		SD	0.172	0.172	0.172	0.176
		MSE	0.030	0.030	0.030	0.031
	$E\left(Y|X_{1}=0,\;X_{2}=0\right)$	bias	−0.007	-	-	-
		SD	0.266	-	-	-
		mean SE	0.262	-	-	-
		MSE	0.071	-	-	-
1000	$\beta_{1}$	bias	0.007	0.007	0.007	0.008
		SD	0.068	0.068	0.068	0.076
		mean SE	0.067	0.067	0.068	0.077
		MSE	0.005	0.005	0.005	0.006
	$\beta_{2}$	bias	−0.001	−0.001	−0.001	−0.001
		SD	0.033	0.033	0.034	0.040
		mean SE	0.034	0.034	0.034	0.041
		MSE	0.001	0.001	0.001	0.002
	$A\left(e^{0.5}\right)$	bias	0.003	0.003	0.003	0.003
		SD	0.055	0.055	0.055	0.056
		mean SE	0.054	0.054	0.054	0.057
		MSE	0.003	0.003	0.003	0.003
	$\text{median}\left(Y|X_{1}=0,\;X_{2}=0\right)$	bias	0.003	0.003	0.002	0.002
		SD	0.054	0.054	0.054	0.056
		MSE	0.003	0.003	0.003	0.003
	$E\left(Y|X_{1}=0,\;X_{2}=0\right)$	bias	−0.003	-	-	-
		SD	0.081	-	-	-
		mean SE	0.083	-	-	-
		MSE	0.007	-	-	-

SD, standard deviation of replicates; mean SE, average estimated standard error across replicates; MSE, mean squared error.

## Data Availability

No new data were created or analyzed in this study. Data sharing is not applicable to this article.

## Appendix A. Proof of Theorem 1

Core steps of the proof: Let $\left(\beta_{0},A_{0}\right)$ be the true value of (β,A) and $\left(\hat{\beta },\hat{A}\right)$ be the NPMLE from the CPM applied to modified data. We will first prove that (I) $\hat{A}$ is bounded in [L,U] with probability one. Since $\hat{A}(\cdot )$ is bounded and increasing in [L,U], via the Helly selection theorem [20], for any subsequence, there exists a weakly convergent subsequence. Thus, without confusion, we assume that $\hat{A}\to A^{*}$ weakly in [L,U] and $\hat{\beta }\to \beta^{*}$. We will then prove that (II) with probability one, $A^{*}(y)=A_{0}(y)$ for y∈[L,U] and $\beta^{*}=\beta_{0}$. With this result, the consistency is established. Furthermore, since $A_{0}$ is continuously differentiable, we conclude that $\hat{A}(y)$ converges to $A_{0}(y)$ uniformly in [L,U] with probability one.

**Proof.** Proof of (I): Given a dataset of i.i.d. observations $\left\{\left(Y_{i},Z_{i}\right)\right\}$, the nonparametric log-likelihood for CPM fitted to the modified data is

$$l_{n}\left(\beta ,A\right)={\text{P}}_{n}\left\{I\left(Y\leq L\right)\text{ log }G\left(A\left(L\right)-\beta^{T}Z\right)+I\left(Y\geq U\right)\text{ log}\left(1-G\left(A\left(U-\right)-\beta^{T}Z\right)\right)+I\left(L<Y<U\right)\text{ log}\left(G\left(A\left(Y\right)-\beta^{T}Z\right)-G\left(A\left(Y-\right)-\beta^{T}Z\right)\right)\right\}.$$

Here, $P_{n}$ denotes the empirical measure, i.e., $P_{n}g(Y,Z)=n^{-1}\sum_{i=1}^{n}\;g\left(Y_{i},Z_{i}\right)$, for any measurable function g(Y,Z). Let $\hat{A}\left\{Y_{i}\right\}\equiv \hat{A}\left(Y_{i}\right)-\hat{A}\left(Y_{i}-\right)$ be the jump size of $\hat{A}$ at $Y_{i}$. Let $\hat{A}(U)\equiv \hat{A}(U-)$.

We first show that lim sup $\hat{A}(U)<\infty$ a.s. If $\hat{A}\left(Y_{i}-\right)\leq M+m$ for all $Y_{i}>0$, then $\hat{A}(U)\leq M+m$. Below, we assume there is a $Y_{i}>0$ such that $\hat{A}\left(Y_{i}-\right)>M+m$. Clearly, $\hat{A}\left\{Y_{i}\right\}$ should be strictly positive, since, otherwise, $l_{n}(\beta ,A)=-\infty$. Because of this, we differentiate $l_{n}(\beta ,A)$ with respect to $A\left\{Y_{i}\right\}$ and then set it to zero to obtain the following equation:

(A1)
$${\text{P}}_{n}\left\{I\left(Y\geq U\right)\frac{G^{\prime }\left(\hat{A}\left(U\right)-{\hat{\beta }}^{T}Z\right)}{1-G\left(\hat{A}\left(U\right)-{\hat{\beta }}^{T}Z\right)}\right\}-{\text{P}}_{n}\left\{I\left(Y_{i}<Y<U\right)\frac{G^{\prime }\left(\hat{A}\left(Y\right)-{\hat{\beta }}^{T}Z\right)-G^{\prime }\left(\hat{A}\left(Y-\right)-{\hat{\beta }}^{T}Z\right)}{G\left(\hat{A}\left(Y\right)-{\hat{\beta }}^{T}Z\right)-G\left(\hat{A}\left(Y-\right)-{\hat{\beta }}^{T}Z\right)}\right\}=\frac{1}{n}\frac{G^{\prime }\left(\hat{A}\left(Y_{i}\right)-{\hat{\beta }}^{T}Z_{i}\right)}{G\left(\hat{A}\left(Y_{i}\right)-{\hat{\beta }}^{T}Z_{i}\right)-G\left(\hat{A}\left(Y_{i}-\right)-{\hat{\beta }}^{T}Z_{i}\right)}.$$

For any $Y>Y_{i}$, via condition 2,

$$\hat{A}\left(Y-\right)-{\hat{\beta }}^{T}Z\geq M+m-{\hat{\beta }}^{T}Z\geq M.$$

According to condition 1, $G^{\prime }(x)$ is decreasing when x≥M. The left-hand side of (A1) is

$$\geq {\text{P}}_{n}\left\{I(Y>U)\frac{G^{\prime }\left(\hat{A}\left(U\right)-{\hat{\beta }}^{T}Z\right)}{1-G\left(\hat{A}\left(U\right)-{\hat{\beta }}^{T}Z\right)}\right\}.$$

For the right-hand side, we use the mean-value theorem on the denominator and then the decreasing property of $G^{\prime }(x)$ when x≥M to obtain that

$$\frac{1}{n}\frac{G^{\prime }\left(\hat{A}\left(Y_{i}\right)-{\hat{\beta }}^{T}Z_{i}\right)}{G\left(\hat{A}\left(Y_{i}\right)-{\hat{\beta }}^{T}Z_{i}\right)-G\left(\hat{A}\left(Y_{i}-\right)-{\hat{\beta }}^{T}Z_{i}\right)}=\frac{1}{n}\frac{G^{\prime }\left(\hat{A}\left(Y_{i}\right)-{\hat{\beta }}^{T}Z_{i}\right)}{G^{\prime }\left(\xi_{i}\right)\hat{A}\left\{Y_{i}\right\}}\leq \frac{1}{n\hat{A}\left\{Y_{i}\right\}},$$

where $\xi_{i}$ is some value such that $\hat{A}\left(Y_{i}-\right)-{\hat{\beta }}^{T}Z_{i}\leq \xi_{i}\leq \hat{A}\left(Y_{i}\right)-{\hat{\beta }}^{T}Z_{i}$. Therefore, we have

$$\hat{A}\left\{Y_{i}\right\}\leq \frac{1}{n}{\left[{\text{P}}_{n}\left\{I(Y>U)\frac{G^{\prime }\left(\hat{A}\left(U\right)-{\hat{\beta }}^{T}Z\right)}{1-G\left(\hat{A}\left(U\right)-{\hat{\beta }}^{T}Z\right)}\right\}\right]}^{-1},$$

and this holds for any $Y_{i}$ between 0 and U and satisfying $\hat{A}\left(Y_{i}-\right)>M+m$.

Let $i_{0}$ be the maximal index i for which $Y_{i}>0$ and $\hat{A}\left(Y_{i_{0}}-\right)\leq M+m$. We sum over all $Y_{i}$ between 0 and U to obtain that

$$\hat{A}\left(U\right)=\hat{A}\left(Y_{i_{0}}-\right)+\sum_{Y_{i}>0,\hat{A}\left(Y_{i}-\right)>M+m}\hat{A}\left\{Y_{i}\right\}\;\leq M+m+\left[n^{-1}\overset{n}{\underset{i=1}{\sum }}I\left(0<Y_{i}\leq U\right)\right]{\left[{\text{P}}_{n}\left\{I(Y>U)\frac{G^{\prime }\left(\hat{A}\left(U\right)-{\hat{\beta }}^{T}Z\right)}{1-G\left(\hat{A}\left(U\right)-{\hat{\beta }}^{T}Z\right)}\right\}\right]}^{-1}.$$

We now show that $\hat{A}(U)$ cannot diverge to ∞. Otherwise, suppose that $\hat{A}(U)\to \infty$ for some subsequence. From the second half of condition 1, when n is large enough in the subsequence, for any Z,

$$\frac{G^{\prime }\left(\hat{A}\left(U\right)-{\hat{\beta }}^{T}Z\right)}{1-G\left(\hat{A}\left(U\right)-{\hat{\beta }}^{T}Z\right)}>\frac{1}{2}\text{ lim }\underset{x\to \infty }{\text{inf}}\frac{G^{\prime }\left(x\right)}{1-G\left(x\right)}\equiv c_{0}>0,$$

and, therefore,

$$\hat{A}\left(U\right)\leq M+m+\frac{n^{-1}\sum_{i=1}^{n}I\left(0<Y_{i}\leq U\right)}{c_{0}{\text{P}}_{n}\{I(Y>U)\}},$$

in which the last term converges to a constant. We thus have a contradiction. Hence, lim sup $\hat{A}(U)<\infty$ with probability 1.

We can reverse the order of $Y_{i}$ (change $Y_{i}$ to $-Y_{i}$ so that the NPMLE is equivalent to maximizing the likelihood function, but instead of A(y), we consider −A(y)). The same arguments as above apply to conclude that lim sup $-\hat{A}(L)<\infty$ with probability 1, or equivalently, lim inf $\hat{A}(L)>-\infty$ with probability 1. □

**Proof.** Proof of (II): We first show that $n\hat{A}\left\{Y_{i}\right\}$ is bounded for all $Y_{i}\in [L,U]$. From the proof above, we know $\hat{A}\left\{Y_{i}\right\}=O\left(n^{-1}\right)$ uniformly in i for which $Y_{i}\in [L,U]$ satisfying $\left|\hat{A}\left(Y_{i}-\right)\right|>M+m$. We prove that this is true for any $Y_{i}$. To do that, we define

$$H_{n}\left(y\right)={\text{P}}_{n}\left\{I(Y>U)\frac{G^{\prime }\left(\hat{A}\left(U\right)-{\hat{\beta }}^{T}Z\right)}{1-G\left(\hat{A}\left(U\right)-{\hat{\beta }}^{T}Z\right)}\right\}-{\text{P}}_{n}\left\{I(y<Y\leq U)\frac{G^{\prime }\left(\hat{A}\left(Y\right)-{\hat{\beta }}^{T}Z\right)-G^{\prime }\left(\hat{A}\left(Y-\right)-{\hat{\beta }}^{T}Z\right)}{G\left(\hat{A}\left(Y\right)-{\hat{\beta }}^{T}Z\right)-G\left(\hat{A}\left(Y-\right)-{\hat{\beta }}^{T}Z\right)}\right\}.$$

First, we note that $H_{n}(y)$ has a total bounded variation in [0,U]. In fact, for any 0<t<s<U,

$$\left|H_{n}\left(t\right)-H_{n}\left(s\right)\right|\;=\left|{\text{P}}_{n}\left\{I(t<Y\leq s)\frac{G^{\prime }\left(\hat{A}\left(Y\right)-{\hat{\beta }}^{T}Z\right)-G^{\prime }\left(\hat{A}\left(Y-\right)-{\hat{\beta }}^{T}Z\right)}{G\left(\hat{A}\left(Y\right)-{\hat{\beta }}^{T}Z\right)-G\left(\hat{A}\left(Y-\right)-{\hat{\beta }}^{T}Z\right)}\right\}\right|\;\leq c_{1}{\text{P}}_{n}\left\{I\left(t<Y\leq s\right)\right\},$$

where $c_{1}={\text{sup}}_{x\in \left[-m,c_{0}+m\right]}\;\left|G^{\prime \prime }(x)\right|/{\text{inf}}_{x\in \left[-m,c_{0}+m\right]}\;\left|G^{\prime }(x)\right|$. By choosing a subsequence, we assume that $H_{n}(y)$ converges weakly to $H^{*}(y)$. From the above inequality and taking limits, it is clear that

$$\left|H^{\text{*}}\left(t\right)-H^{\text{*}}\left(s\right)\right|\leq c_{1}P(t<Y\leq s),$$

so $H^{*}(y)$ is Lipschitz-continuous in y∈[0,U]. The latter property ensures that $H_{n}(y)$ uniformly converges to $H^{*}(t)$ for t∈[0,U].

According to Equation (A1), we know

(A2)
$$\left|H_{n}\left(Y_{i}\right)\right|=\frac{1}{n}\frac{G^{\prime }\left(\hat{A}\left(Y_{i}\right)-{\hat{\beta }}^{T}Z_{i}\right)}{G\left(\hat{A}\left(Y_{i}\right)-{\hat{\beta }}^{T}Z_{i}\right)-G\left(\hat{A}\left(Y_{i}-\right)-{\hat{\beta }}^{T}Z_{i}\right)}\geq \frac{c_{2}}{n\hat{A}\left\{Y_{i}\right\}},$$

where $c_{2}={\text{inf}}_{x\in \left[-m,c_{0}+m\right]G^{\prime }(x)}\;/{\text{sup}}_{x\in \left[-m,c_{0}+m\right]}\;G^{\prime }(x)$. Thus,

$$\hat{A}\left\{Y_{i}\right\}\geq \frac{c_{2}}{n}\frac{1}{\left|H_{n}\left(Y_{i}\right)\right|+\epsilon }$$

for any positive constant ϵ. This gives

(A3)
$$\hat{A}\left(U\right)\geq c_{2}{\text{P}}_{n}\left[\frac{I\left(Y\in \left[0,U\right]\right)}{\left|H_{n}\left(Y\right)\right|+\epsilon }\right].$$

Since $H_{n}(Y)$ has a bounded total variation, ${\left\{\left|H_{n}(Y)\right|+\epsilon \right\}}^{-1}$ belongs to a Glivenko–Cantelli class bounded by 1/ϵ and it converges in $L_{2}(P)$-norm to ${\left\{\left|H^{*}(Y)\right|+\epsilon \right\}}^{-1}$. As a result, the right-hand side of (A3) converges to $c_{2}E\left[I(Y\in [0,U]){\left(\left|H^{*}(Y)\right|+\epsilon \right)}^{-1}\right]$, so we obtain that

$$c_{0}\geq c_{2}\int_{0}^{U}\frac{f_{Y}\left(y\right)}{\left|H^{\text{*}}\left(y\right)\right|+\epsilon }dy,$$

where $f_{Y}(y)$ is the marginal density of Y. Let ϵ decrease to zero; then, from the monotone convergence theorem, we conclude that

(A4)
$$\int_{0}^{U}\frac{f_{Y}\left(y\right)}{\left|H^{\text{*}}\left(y\right)\right|}dy\leq \frac{c_{0}}{c_{2}}.$$

We use (A4) to show that ${\text{min}}_{y\in [0,\tau ]}\;\left|H^{*}(y)\right|>0$. Otherwise, since $H^{*}(y)$ is continuous, there exists some $y_{0}\in [0,\tau ]$ such that $H^{*}\left(y_{0}\right)=0$. However, since $H^{*}(y)$ is Lipschitz-continuous at $y_{0}$, the left-hand side of (A4) is at least larger than $\int_{y_{0}}^{y_{0}+\delta }\;{\left\{c_{1}\left|y-y_{0}\right|\right\}}^{-1}dy$ if $y_{0}<U$ or $\int_{y_{0}-\delta }^{y_{0}}\;{\left\{c_{1}\left|y-y_{0}\right|\right\}}^{-1}dy$ if $y_{0}>0$ for some small constant δ. The latter integrals are infinity. We obtain the contradiction. Hence, we conclude that $H^{*}(y)$ is uniformly bounded away from zero when y∈[0,U]. Thus, when n is large enough, $\left|{\hat{H}}_{n}\left(Y_{i}\right)\right|$ is larger than a positive constant $c_{3}$ uniformly for all $Y_{i}>0$. From (A1), we thus obtain that

$$c_{3}\leq \frac{c_{4}}{n\hat{A}\left\{Y_{i}\right\}},$$

where $c_{4}={\text{sup}}_{x\in \left[-m,c_{0}+m\right]G^{\prime }(x)}\;/{\text{inf}}_{x\in \left[-m,c_{0}+m\right]}\;G^{\prime }(x)$. In other words, $n\hat{A}\left\{Y_{i}\right\}\leq c_{4}/c_{3}$. Using symmetric arguments, we can show that $n\hat{A}\left\{Y_{i}\right\}$ is bounded by a constant for all $Y_{i}<0$.

Finally, to establish the consistency in Theorem 1, since $\hat{A}\left\{Y_{i}\right\}$ is of order $n^{-1}$, from Equation (A1), we obtain that

$$\hat{A}\left\{Y_{i}\right\}=n^{-1}{\left[{\text{P}}_{n}\left\{I(Y>U)\frac{G^{\prime }\left(\hat{A}\left(U\right)-{\hat{\beta }}^{T}Z\right)}{1-G\left(\hat{A}\left(U\right)-{\hat{\beta }}^{T}Z\right)}\right\}-{\text{P}}_{n}\left\{I\left(Y_{i}<Y\leq U\right)\frac{G^{\prime \prime }\left(\hat{A}\left(Y\right)-{\hat{\beta }}^{T}Z\right)}{G^{\prime }\left(\hat{A}\left(Y\right)-{\hat{\beta }}^{T}Z\right)}\right\}\right]}^{-1}+O\left(n^{-2}\right).$$

Following this expression, we define another step function, denoted by $\tilde{A}(y)$, whose jump size at $Y_{i}$ satisfies that

$$\tilde{A}\left\{Y_{i}\right\}=n^{-1}{\left[{\text{P}}_{n}\left\{I(Y>U)\frac{G^{\prime }\left(A_{0}\left(U\right)-\beta_{0}^{T}Z\right)}{1-G\left(A_{0}\left(U\right)-\beta_{0}^{T}Z\right)}\right\}-{\text{P}}_{n}\left\{I\left(Y_{i}<Y\leq U\right)\frac{G^{\prime \prime }\left(A_{0}\left(Y\right)-\beta_{0}^{T}Z\right)}{G^{\prime }\left(A_{0}\left(Y\right)-\beta_{0}^{T}Z\right)}\right\}\right]}^{-1}$$

so

$$\tilde{A}\left(y\right)=\;n^{-1}\sum_{i=1}^{n}I\left(Y_{i}\leq y\right){\left[{\text{P}}_{n}\left\{I(Y>U)\frac{G^{\prime }\left(A_{0}\left(U\right)-\beta_{0}^{T}Z\right)}{1-G\left(A_{0}\left(U\right)-\beta_{0}^{T}Z\right)}\right\}-{\text{P}}_{n}\left\{I\left(Y_{i}<Y\leq U\right)\frac{G^{\prime \prime }\left(A_{0}\left(Y\right)-\beta_{0}^{T}Z\right)}{G^{\prime }\left(A_{0}\left(Y\right)-\beta_{0}^{T}Z\right)}\right\}\right]}^{-1}.$$

Via the strong law of large numbers and monotonicity of $\hat{A}$, it is straightforward to show that $\hat{A}(y)$ converges to

$$E\left\{I\left(Y\leq y\right){\left[\tilde{\text{P}}\left\{I(\tilde{Y}>U)\frac{G^{\prime }\left(A_{0}\left(U\right)-\beta_{0}^{T}\tilde{Z}\right)}{1-G\left(A_{0}\left(U\right)-\beta_{0}^{T}\tilde{Z}\right)}\right\}-\tilde{\text{P}}\left\{I(Y<\tilde{Y}\leq U)\frac{G^{\prime \prime }\left(A_{0}\left(\tilde{Y}\right)-\beta_{0}^{T}\tilde{Z}\right)}{G^{\prime }\left(A_{0}\left(\tilde{Y}\right)-\beta_{0}^{T}\tilde{Z}\right)}\right\}\right]}^{-1}\right\}$$

uniformly in y∈[L,U]. The limit can be verified to be the same as $A_{0}(y)$. Furthermore, we notice

(A5)
$$\frac{\hat{A}\left\{Y_{i}\right\}}{\tilde{A}\left\{Y_{i}\right\}}=\frac{{\text{P}}_{n}\left\{I(Y>U)\frac{G^{\prime }\left(A_{0}\left(U\right)-\beta_{0}^{T}Z\right)}{1-G\left(A_{0}\left(U\right)-\beta_{0}^{T}Z\right)}\right\}-{\text{P}}_{n}\left\{I\left(Y_{i}<Y\leq U\right)\frac{G^{\prime \prime }\left(A_{0}\left(Y\right)-\beta_{0}^{T}Z\right)}{G^{\prime }\left(A_{0}\left(Y\right)-\beta_{0}^{T}Z\right)}\right\}}{\left[{\text{P}}_{n}\left\{I(Y>U)\frac{G^{\prime }\left(\hat{A}\left(U\right)-{\hat{\beta }}^{T}Z\right)}{1-G\left(\hat{A}\left(U\right)-{\hat{\beta }}^{T}Z\right)}\right\}-{\text{P}}_{n}\left\{I\left(Y_{i}<Y\leq U\right)\frac{G^{\prime \prime }\left(\hat{A}\left(Y\right)-{\hat{\beta }}^{T}Z\right)}{G^{\prime }\left(\hat{A}\left(Y\right)-{\hat{\beta }}^{T}Z\right)}\right\}\right]+O\left(n^{-1}\right)}.$$

Since $\hat{A}(y)$ is bounded and increasing and ${\hat{\beta }}^{T}Z,\hat{A}(Y)-{\hat{\beta }}^{T}Z$ belongs to a VC-hull of Donsker class. Via the preservation property under the monotone transformation, $G^{\left(k\right)}\left(\hat{A}(U)-{\hat{\beta }}^{T}Z\right),k=0,1,2$, also belongs to a Donsker class. Therefore, the right-hand side of (A5) converges uniformly in $Y_{i}$ to

$$g\left(Y_{i}\right)=\frac{\text{P}\left\{I(Y>U)\frac{G^{\prime }\left(A_{0}\left(U\right)-\beta_{0}^{T}Z\right)}{1-G\left(A_{0}\left(U\right)-\beta_{0}^{T}Z\right)}\right\}-\text{P}\left\{I\left(Y_{i}<Y\leq U\right)\frac{G^{\prime \prime }\left(A_{0}\left(Y\right)-\beta_{0}^{T}Z\right)}{G^{\prime }\left(A_{0}\left(Y\right)-\beta_{0}^{T}Z\right)}\right\}}{\text{P}\left\{I(Y>U)\frac{G^{\prime }\left(A^{\text{*}}\left(U\right)-\beta^{\text{*}T}Z\right)}{1-G\left(A^{\text{*}}\left(U\right)-\beta^{\text{*}T}Z\right)}\right\}-\text{P}\left\{I\left(Y_{i}<Y\leq U\right)\frac{G^{\prime \prime }\left(A^{\text{*}}\left(Y\right)-\beta^{\text{*}T}Z\right)}{G^{\prime }\left(A^{\text{*}}\left(Y\right)-\beta^{\text{*}T}Z\right)}\right\}}.$$

As a result, $A^{*}(y)=\int_{0}^{y}\;g(t)dA_{0}(t)$, or, equivalently, $dA^{*}(y)/dA_{0}(y)=g(y)$.

Define

$${\tilde{l}}_{n}\left(\beta ,A\right)={\text{P}}_{n}\left\{I\left(Y\leq L\right)\text{ log }G\left(A\left(L\right)-\beta^{T}Z\right)+I(Y>U)\text{ log}\left(1-G\left(A\left(U\right)-\beta^{T}Z\right)\right)+I(L<Y\leq U)\text{ log}(G\left(A\left(Y\right)-\beta^{T}Z\right)A\left\{Y\right\}\right\}.$$

Since $\tilde{A}\left\{Y_{i}\right\}=O\left(n^{-1}\right)$ and $\hat{A}\left\{Y_{i}\right\}=O\left(n^{-1}\right)$,

$$l_{n}\left(\hat{\beta },\hat{A}\right)={\tilde{l}}_{n}\left(\hat{\beta },\hat{A}\right)+O\left(n^{-1}\right),l_{n}\left(\beta_{0},\tilde{A}\right)={\tilde{l}}_{n}\left(\beta_{0},\tilde{A}\right)+O\left(n^{-1}\right).$$

Since $l_{n}(\hat{\beta },\hat{A})\geq l_{n}\left(\beta_{0},{\tilde{A}}_{0}\right)$, we have

$${\tilde{l}}_{n}\left(\hat{\beta },\hat{A}\right)\geq {\tilde{l}}_{n}\left(\beta_{0},\tilde{A}\right)+O\left(n^{-1}\right).$$

That is,

$${\text{P}}_{n}\left\{I\left(Y\leq L\right)\text{ log}\frac{G\left(\hat{A}\left(L\right)-{\hat{\beta }}^{T}Z\right)}{G\left(\tilde{A}\left(L\right)-\beta_{0}^{T}Z\right)}+I\left(Y>U\right)\text{ log}\frac{1-G\left(\hat{A}\left(U\right)-{\hat{\beta }}^{T}Z\right)}{1-G\left(\tilde{A}\left(U\right)-\beta_{0}^{T}Z\right)}\right\}\;+{\text{P}}_{n}\left\{I\left(L<Y\leq U\right)\text{ log}\frac{G\left(\hat{A}\left(Y\right)-{\hat{\beta }}^{T}Z\right)}{G\left(\tilde{A}\left(Y\right)-\beta_{0}^{T}Z\right)}\right\}+n^{-1}\overset{n}{\underset{i=1}{\sum }}I\left(L<Y_{i}\leq U\right)\frac{\hat{A}\left\{Y_{i}\right\}}{\tilde{A}\left\{Y_{i}\right\}}\geq O\left(n^{-1}\right).$$

We take limits on both sides. Using the Glivenko–Cantelli theorem with the first three two terms in the left-hand side and noting $\left|\hat{A}\left\{Y_{i}\right\}/\tilde{A}\left\{Y_{i}\right\}-g\left(Y_{i}\right)\right|$ converges to zero uniformly, we obtain that

$$\text{P}\left\{I\left(Y\leq L\right)\text{ log}\frac{G\left(A^{\text{*}}\left(L\right)-\beta^{\text{*}T}Z\right)}{G\left(A_{0}\left(L\right)-\beta_{0}^{T}Z\right)}+I\left(Y>U\right)\text{ log}\frac{1-G\left(A^{\text{*}}\left(U\right)-\beta^{\text{*}T}Z\right)}{1-G\left(A_{0}\left(U\right)-\beta_{0}^{T}Z\right)}\right\}\;+\text{P}\left\{I\left(L<Y\leq U\right)\text{ log}\frac{G\left(A^{\text{*}}\left(Y\right)-\beta^{\text{*}T}Z\right)}{G\left(A_{0}\left(Y\right)-\beta_{0}^{T}Z\right)}\right\}+\text{P}\left\{(L<Y\leq U)\frac{dA^{\text{*}}\left(Y\right)}{dA_{0}\left(Y\right)}\right\}\geq 0.$$

The left-hand side is the negative Kullback–Leibler information for the density with parameter $\left(\beta^{*},A^{*}\right)$. Thus, the density function with parameter $\left(\beta^{*},A^{*}\right)$ should be the same as the true density. Immediately, we obtain that

$$G\left(A^{\text{*}}\left(Y\right)+\beta^{\text{*}T}Z\right)=G\left(A_{0}\left(Y\right)+\beta_{0}^{T}Z\right)$$

with probability one. From condition 2, we conclude that $\beta^{*}=\beta_{0}$ and $A^{*}(y)=A_{0}(y)$ for y∈[L,U]. □

## Appendix B. Proof of Theorem 2

**Proof.** Let BV[L,U] be the set of the functions over [L,U] with $∥h{∥}_{TV}\leq 1$, where $∥\cdot {∥}_{TV}$ denotes the total variation in [L,U]. For any $v\in R^{p}$ with ∥v∥ ≤1 and any h∈BV[L,U], we define the score function $\Psi_{n}(\beta ,A)[v,h]$ along the submodel for β with tangent direction v and for A with the tangent function $\int_{0}^{.}\;h(t)dA(t)$:

$$\Psi_{n}\left(\beta ,A\right)\left[v,h\right]\;=\underset{\epsilon \to 0}{\text{lim}}\left(\frac{l_{n}\left(\beta +\epsilon v,A+\epsilon \;\int_{0}^{\cdot }h\left(t\right)dA\left(t\right)\right)-l_{n}\left(\beta ,A\right)}{\epsilon }\right)\;={\text{P}}_{n}\left\{-F_{1}\left(Y,Z;\beta ,A\right)Z^{T}v+F_{2}\left(Y,Z;\beta ,A\right)Z^{T}v-F_{3}\left(Y,Z;\beta ,A\right)Z^{T}v\right\}+{\text{P}}_{n}\left\{F_{1}\left(Y,Z;\beta ,A\right)\int_{0}^{L}h\left(t\right)dA\left(t\right)-F_{2}\left(Y,Z;\beta ,A\right)\int_{0}^{U}h\left(t\right)dA\left(t\right)+F_{3}\left(Y,Z;\beta ,A\right)\int_{0}^{Y}hdA+F_{4}\left(Y,Z;\beta ,A\right)h\left(Y\right)\right\},$$

where

$$\begin{array}{c} F_{1}\left(Y,Z;\beta ,A\right)=\frac{I\left(Y\leq L\right)G^{\prime }\left(A\left(L\right)-\beta^{T}Z\right)}{G\left(A\left(L\right)-\beta^{T}Z\right)}, \end{array}$$

$$\begin{array}{c} F_{2}\left(Y,Z;\beta ,A\right)=\frac{I\left(Y\geq U\right)G^{\prime }\left(A\left(U\right)-\beta^{T}Z\right)}{1-G\left(A\left(U\right)-\beta^{T}Z\right)}, \end{array}$$

$$\begin{array}{c} F_{3}\left(Y,Z;\beta ,A\right)=\frac{I(L<Y<U)\left(G^{\prime }\left(A\left(Y\right)-\beta^{T}Z\right)-G^{\prime }\left(A\left(Y-\right)-\beta^{T}Z\right)\right)}{G\left(A\left(Y\right)-\beta^{T}Z\right)-G\left(A\left(Y-\right)-\beta^{T}Z\right)}, \end{array}$$

$$\begin{array}{c} F_{4}\left(Y,Z;\beta ,A\right)=\frac{G^{\prime }\left(A\left(Y-\right)-\beta^{T}Z\right)}{G\left(A\left(Y\right)-\beta^{T}Z\right)-G\left(A\left(Y-\right)-\beta^{T}Z\right)}\left(A\left(Y\right)-A\left(Y-\right)\right). \end{array}$$

Since $\left(\hat{\beta },\hat{A}\right)$ maximizes $l_{n}(\beta ,A)$, we have, for any v and h,

$$\Psi_{n}\left(\hat{\beta },\hat{A}\right)\left[v,h\right]=0.$$

The rest of the proof contains the following main steps: we first show that $\left(\hat{\beta },\hat{A}\right)$ satisfies Equation (A6) (details below), then (A8), and finally (A10), from which the asymptotic distribution of $\left(\hat{\beta },\hat{A}\right)$ will be derived.

We know ${\text{max}}_{L\leq Y_{i}\leq U}\;\left(\hat{A}\left(Y_{i}\right)-\hat{A}\left(Y_{i}-\right)\right)=O_{p}\left(n^{-1}\right)$ from the proof in Appendix A. Thus, if we let

$${\tilde{F}}_{3}\left(Y,Z;\beta ,A\right)=\frac{I(L<Y<U)G^{\prime \prime }\left(A\left(Y\right)-\beta^{T}Z\right)}{G^{\prime }\left(A\left(Y\right)-\beta^{T}Z\right)},$$

then

$$F_{3}\left(Y,Z;\hat{\beta },\hat{A}\right)={\tilde{F}}_{3}\left(Y,Z;\hat{\beta },\hat{A}\right)+O_{p}\left(n^{-1}\right)$$

and $F_{4}(Y,Z;\hat{\beta },\hat{A})=1+O_{p}\left(n^{-1}\right)$ uniformly in (Y,Z). Consequently, we obtain that

$${\text{P}}_{n}\left(-F_{1}\left(Y,Z;\hat{\beta },\hat{A}\right)Z^{T}v+F_{2}\left(Y,Z;\hat{\beta },\hat{A}\right)Z^{T}v-{\tilde{F}}_{3}\left(Y,Z;\hat{\beta },\hat{A}\right)Z^{T}v\right)\;+{\text{P}}_{n}\left(F_{1}\left(Y,Z;\hat{\beta },\hat{A}\right)\int_{0}^{L}h\left(t\right)d\hat{A}\left(t\right)-F_{2}\left(Y,Z;\hat{\beta },\hat{A}\right)\int_{0}^{U}h\left(t\right)d\hat{A}\left(t\right)+{\tilde{F}}_{3}\left(Y,Z;\hat{\beta },\hat{A}\right)\int_{0}^{Y}hd\hat{A}+h\left(Y\right)\right)\;=O_{p}\left(n^{-1}\right),$$

and it holds uniformly in v and h with ∥v∥ ≤1 and $∥h{∥}_{TV}\leq 1$. Equivalently, we have that

(A6)
$$\sqrt{n}\left({\text{P}}_{n}-\text{P}\right)\left(\begin{array}{c} -F_{1}\left(Y,Z;\hat{\beta },\hat{A}\right)Z^{T}v+F_{2}\left(Y,Z;\hat{\beta },\hat{A}\right)Z^{T}v-{\tilde{F}}_{3}\left(Y,Z;\hat{\beta },\hat{A}\right)Z^{T}v\\ +F_{1}\left(Y,Z;\hat{\beta },\hat{A}\right)\int_{0}^{L}h\left(t\right)d\hat{A}\left(t\right)-F_{2}\left(Y,Z;\hat{\beta },\hat{A}\right)\int_{0}^{U}h\left(t\right)d\hat{A}\left(t\right)\\ +{\tilde{F}}_{3}\left(Y,Z;\hat{\beta },\hat{A}\right)\int_{0}^{Y}hd\hat{A}+h\left(Y\right) \end{array}\right)\;=\sqrt{n}\text{P}\left(\begin{array}{c} -F_{1}\left(Y,Z;\hat{\beta },\hat{A}\right)Z^{T}v+F_{2}\left(Y,Z;\hat{\beta },\hat{A}\right)Z^{T}v-{\tilde{F}}_{3}\left(Y,Z;\hat{\beta },\hat{A}\right)Z^{T}v\\ +F_{1}\left(Y,Z;\hat{\beta },\hat{A}\right)\int_{0}^{L}h\left(t\right)d\hat{A}\left(t\right)-F_{2}\left(Y,Z;\hat{\beta },\hat{A}\right)\int_{0}^{U}h\left(t\right)d\hat{A}\left(t\right)\\ +{\tilde{F}}_{3}\left(Y,Z;\hat{\beta },\hat{A}\right)\int_{0}^{Y}hd\hat{A}+h\left(Y\right) \end{array}\right)+O_{p}(n^{\left(-1/2\right)}).$$

For the left-hand side of (A6), it is easy to see that for (β,A) in a neighborhood of $\left(\beta_{0},A_{0}\right)$ in the metric space $R^{d}\times BV[L,U]$, the classes of $F_{1}(Y,Z;\beta ,A),F_{2}(Y,Z;\beta ,A)$ and ${\tilde{F}}_{3}(Y,Z;\beta ,A)$ are Lipschtiz classes of the P-Donsker classes $\left\{\beta^{T}Z\right\}$ and {A(Y)∈BV[L,U]}, so they are P-Donsker by preservation of the Donsker property. Additionally, the classes of $\left\{\int_{0}^{Y}\;hdA\right\},\left\{Z^{T}v\right\}$, and {h(Y)} are P-Donsker. As the result, since, via the consistency,

$$\left(\begin{array}{c} -F_{1}\left(Y,Z;\hat{\beta },\hat{A}\right)Z^{T}v+F_{2}\left(Y,Z;\hat{\beta },\hat{A}\right)Z^{T}v-{\tilde{F}}_{3}\left(Y,Z;\hat{\beta },\hat{A}\right)Z^{T}v\\ +F_{1}\left(Y,Z;\hat{\beta },\hat{A}\right)\int_{0}^{L}h\left(t\right)d\hat{A}\left(t\right)-F_{2}\left(Y,Z;\hat{\beta },\hat{A}\right)\int_{0}^{U}h\left(t\right)d\hat{A}\left(t\right)\\ +{\tilde{F}}_{3}\left(Y,Z;\hat{\beta },\hat{A}\right)\int_{0}^{Y}hd\hat{A}+h\left(Y\right) \end{array}\right)$$

converges in $L_{2}(P)$ to

$$\text{S}\left(Y,Z\right)\left[v,h\right]\equiv \left(\begin{array}{c} -F_{1}\left(Y,Z;\beta_{0},A_{0}\right)Z^{T}v+F_{2}\left(Y,Z;\beta_{0},A_{0}\right)Z^{T}v-{\tilde{F}}_{3}\left(Y,Z;\beta_{0},A_{0}\right)Z^{T}v\\ +F_{1}\left(Y,Z;\beta_{0},A_{0}\right)\int_{0}^{L}h\left(t\right)dA_{0}\left(t\right)-F_{2}\left(Y,Z;\beta_{0},A_{0}\right)\int_{0}^{U}h\left(t\right)dA_{0}\left(t\right)\\ +{\tilde{F}}_{3}\left(Y,Z;\beta_{0},A_{0}\right)\int_{0}^{Y}hdA_{0}+h\left(Y\right) \end{array}\right),$$

Equation (A6) gives that

(A7)
$$\sqrt{n}\left({\text{P}}_{n}-\text{P}\right)\text{S}\left(Y,Z\right)\left[v,h\right]\;=\sqrt{n}\text{P}\left(\begin{array}{c} -F_{1}\left(Y,Z;\hat{\beta },\hat{A}\right)Z^{T}v+F_{2}\left(Y,Z;\hat{\beta },\hat{A}\right)Z^{T}v-{\tilde{F}}_{3}\left(Y,Z;\hat{\beta },\hat{A}\right)Z^{T}v\\ F_{1}\left(Y,Z;\hat{\beta },\hat{A}\right)\int_{0}^{L}h\left(t\right)d\hat{A}\left(t\right)-F_{2}\left(Y,Z;\hat{\beta },\hat{A}\right)\int_{0}^{U}h\left(t\right)d\hat{A}\left(t\right)\\ +{\tilde{F}}_{3}\left(Y,Z;\hat{\beta },\hat{A}\right)\int_{0}^{Y}hd\hat{A}+h\left(Y\right) \end{array}\right)+o_{p}\left(1\right).$$

On the other hand, we note that the first term in the right-hand side of (A7) is zero if replacing $\left(\hat{\beta },\hat{A}\right)$ by $\left(\beta_{0},A_{0}\right)$. Thus, the right-hand side of (A7) is equal to

$$\sqrt{n}\text{P}\left(\begin{array}{c} -F_{1}\left(Y,Z;\hat{\beta },\hat{A}\right)Z^{T}v+F_{2}\left(Y,Z;\hat{\beta },\hat{A}\right)Z^{T}v-{\tilde{F}}_{3}\left(Y,Z;\hat{\beta },\hat{A}\right)Z^{T}v\\ +F_{1}\left(Y,Z;\hat{\beta },\hat{A}\right)\int_{0}^{L}h\left(t\right)d\hat{A}\left(t\right)-F_{2}\left(Y,Z;\hat{\beta },\hat{A}\right)\int_{0}^{U}h\left(t\right)d\hat{A}\left(t\right)\\ +{\tilde{F}}_{3}\left(Y,Z;\hat{\beta },\hat{A}\right)\int_{0}^{Y}hd\hat{A} \end{array}\right)$$

$$-\sqrt{n}\text{P}\left(\begin{array}{c} -F_{1}\left(Y,Z;\beta_{0},A_{0}\right)Z^{T}v+F_{2}\left(Y,Z;\beta_{0},A_{0}\right)Z^{T}v-{\tilde{F}}_{3}\left(Y,Z;\beta_{0},A_{0}\right)Z^{T}v\\ +F_{1}\left(Y,Z;\beta_{0},A_{0}\right)\int_{0}^{L}h\left(t\right)dA_{0}\left(t\right)-F_{2}\left(Y,Z;\beta_{0},A_{0}\right)\int_{0}^{U}h\left(t\right)dA_{0}\left(t\right)\\ +{\tilde{F}}_{3}\left(Y,Z;\beta_{0},A_{0}\right)\int_{0}^{Y}hdA_{0} \end{array}\right)+o_{p}\left(1\right).$$

We perform the linearization to the first two terms in the above expression. After some algebra, we obtain that this expression is equivalent to

$$\sqrt{n}{\left({\text{S}}_{11}^{T}v+{\text{S}}_{12}\left[h\right]\right)}^{T}\left(\hat{\beta }-\beta_{0}\right)+\sqrt{n}\int \;\left({\text{S}}_{21}^{T}v+{\text{S}}_{22}\left[h\right]\right)d\left(\hat{A}-A_{0}\right)\left(y\right)\;\;+o_{p}\left(\sqrt{n}\left\|\hat{\beta }-\beta_{0}\right\|+\sqrt{n}{\left\|\hat{A}-A_{0}\right\|}_{TV}\right)+o_{p}\left(1\right),$$

where the operators $S_{11}:R^{d}\to R^{d},S_{12}:BV[L,U]\to R^{d},S_{21}^{T}:R^{d}\to BV[L,U]$, and $S_{22}:BV[L,U]\to BV[L,U]$ are defined as

$${\text{S}}_{11}v=E\left[{\frac{d}{dt}\frac{G^{\prime }\left(t\right)}{G\left(t\right)}|}_{t=A_{0}\left(L\right)-\beta_{0}^{T}Z}I\left(Y\leq L\right)ZZ^{T}v\right]\;-E\left[{\frac{d}{dt}\frac{G^{\prime }\left(t\right)}{1-G\left(t\right)}|}_{t=A_{0}\left(U\right)-\beta_{0}^{T}Z}I\left(Y\geq U\right)ZZ^{T}v\right]\;+E\left[{\frac{d}{dt}\frac{G^{\prime \prime }\left(t\right)}{G^{\prime }\left(t\right)}|}_{t=A_{0}\left(Y\right)-\beta_{0}^{T}Z}I(L<Y<U)ZZ^{T}v\right],$$

$${\text{S}}_{12}\left[h\right]=-E\left[{\frac{d}{dt}\frac{G^{\prime }\left(t\right)}{G\left(t\right)}|}_{t=A_{0}\left(L\right)-\beta_{0}^{T}Z}I\left(Y\leq L\right)Z\int_{0}^{L}hdA_{0}\right]\;+E\left[{\frac{d}{dt}\frac{G^{\prime }\left(t\right)}{1-G\left(t\right)}|}_{t=A_{0}\left(U\right)-\beta_{0}^{T}Z}I\left(Y\geq U\right)Z\int_{0}^{U}hdA_{0}\right]\;-E\left[{\frac{d}{dt}\frac{G^{\prime \prime }\left(t\right)}{G^{\prime }\left(t\right)}|}_{t=A_{0}\left(Y\right)-\beta_{0}^{T}Z}I(L<Y<U)Z\int_{0}^{Y}hdA_{0}\right],$$

$$\left({\text{S}}_{21}^{T}v\right)\left(y\right)=-E\left[{\frac{d}{dt}\frac{G^{\prime }\left(t\right)}{G\left(t\right)}|}_{t=A_{0}\left(L\right)-\beta_{0}^{T}Z}I\left(Y\leq L\right)Z^{T}vI(Y>y)\right]\;+E\left[{\frac{d}{dt}\frac{G^{\prime }\left(t\right)}{1-G\left(t\right)}|}_{t=A_{0}\left(U\right)-\beta_{0}^{T}Z}I\left(Y\geq U\right)Z^{T}vI(Y>y)\right]\;-E\left[{\frac{d}{dt}\frac{G^{\prime \prime }\left(t\right)}{G^{\prime }\left(t\right)}|}_{t=A_{0}\left(Y\right)-\beta_{0}^{T}Z}I(L<Y<U)Z^{T}vI(Y>y)\right],$$

$${\text{S}}_{22}\left[h\right]\left(y\right)=E\left[{\frac{d}{dt}\frac{G^{\prime }\left(t\right)}{G\left(t\right)}|}_{t=A_{0}\left(L\right)-\beta_{0}^{T}Z}I\left(Y\leq L\right)I(Y>y)\int_{0}^{L}hdA_{0}\right]\;-E\left[{\frac{d}{dt}\frac{G^{\prime }\left(t\right)}{1-G\left(t\right)}|}_{t=A_{0}\left(U\right)-\beta_{0}^{T}Z}I\left(Y\geq U\right)I(Y>y)\int_{0}^{U}hdA_{0}\right]\;+E\left[{\frac{d}{dt}\frac{G^{\prime \prime }\left(t\right)}{G^{\prime }\left(t\right)}|}_{t=A_{0}\left(Y\right)-\beta_{0}^{T}Z}I(L<Y<U)I(Y>y)\int_{0}^{Y}hdA_{0}\right]\;+E\left\{F_{1}\left(Y,Z;\beta_{0},A_{0}\right)I\left(L\geq y\right)-F_{2}\left(Y,Z;\beta_{0},A_{0}\right)I(U>y)+{\tilde{F}}_{3}\left(Y,Z;\beta_{0},A_{0}\right)I\left(Y\leq y\right)\right\}h\left(y\right).$$

Combining the above results, we obtain from (A7) that

(A8)
$$\sqrt{n}{\left({\text{S}}_{11}^{T}v+{\text{S}}_{12}\left[h\right]\right)}^{T}\left(\hat{\beta }-\beta_{0}\right)+\sqrt{n}\int \;\left({\text{S}}_{21}^{T}v+{\text{S}}_{22}\left[h\right]\right)d\left(\hat{A}-A_{0}\right)\left(y\right)\;=\sqrt{n}\left({\text{P}}_{n}-\text{P}\right)\text{S}\left(Y,Z\right)\left[v,h\right]+o_{p}\left(\sqrt{n}\left\|\hat{\beta }-\beta_{0}\right\|+\sqrt{n}{\left\|\hat{A}-A_{0}\right\|}_{TV}\right)+o_{p}\left(1\right).$$

Next, we show that the operator, $\left(S_{11}^{T}v+S_{12}[h],S_{21}^{T}v+S_{22}[h]\right)$ that maps $\left(v,h)\in R^{d}\times BV[L,U\right]$ to $R^{d}\times BV[L,U]$, is invertible. This can be proven as follows: first, $S_{11}^{T}v+S_{12}[h]$ is finite-dimensional. Second, since the last term of $S_{22}[h]$ is invertible and the other terms in $S_{21}^{T}v+S_{22}[h]$ map (v,h) to a continuously differentiable function in [L,U] which is a compact operator, $S_{21}^{T}v+S_{22}[h]$ is a Fredholm operator of the first kind. Therefore, to prove the invertibility, it suffices to show that $\left(S_{11}^{T}v+S_{12}[h],S_{21}^{T}v+S_{22}[h]\right)$ is one-to-one. Suppose that $\left(S_{11}^{T}v+S_{12}[h],S_{21}^{T}v+S_{22}[h]\right)=0$. Thus, we have

$${\left({\text{S}}_{11}^{T}v+{\text{S}}_{12}\left[h\right]\right)}^{T}v+\int \;\left({\text{S}}_{21}^{T}v+{\text{S}}_{22}\left[h\right]\right)dh\left(y\right)=0.$$

From the previous derivation, we note that the left-hand side is in fact the negative Fisher information along the submodel $\left(\beta_{0}+\epsilon v,A_{0}+\int_{0}^{.}\;\;h(t)dA_{0}(t)\right)$. Thus, the score function along this submodel must almost certainly be zero. That is,

$$-F_{1}\left(Y,Z;\beta_{0},A_{0}\right)Z^{T}v+F_{2}\left(Y,Z;\beta_{0},A_{0}\right)Z^{T}v-F_{3}\left(Y,Z;\beta_{0},A_{0}\right)Z^{T}v\;+F_{1}\left(Y,Z;\beta_{0},A_{0}\right)\int_{0}^{L}h\left(t\right)dA\left(t\right)-F_{2}\left(Y,Z;\beta_{0},A_{0}\right)\int_{0}^{U}h\left(t\right)dA\left(t\right)\;+{\tilde{F}}_{3}\left(Y,Z;\beta_{0},A_{0}\right)\int_{0}^{Y}hdA+h\left(Y\right)=0$$

almost certainly. Consider any Y=0; then, we have $Z^{T}v-h(0)=0$, so v=0 from condition 2. This further shows that h(0)=0 and h(y) satisfies

$$h\left(Y\right)+{\tilde{F}}_{3}\left(Y,Z;\beta_{0},A_{0}\right)\int_{0}^{Y}hdA=0$$

for any Y∈[L,U]. This is a homogeneous integral equation and it is clear h(y)=0 for any y∈[L,U]. We thus have established the invertibility of the operator $\left(S_{11}^{T}v+S_{12}[h],S_{21}^{T}v+\left.S_{22}[h]\right)\right.$.

Therefore, from (A8), for any $v^{*}$ and $h^{*}$, by choosing $\left(v^{-},h^{-}\right)$ as the inverse of the above operator applied to $\left(v^{*},h^{*}\right)$, we obtain that

(A9)
$$\sqrt{n}v^{\text{*}T}\left(\hat{\beta }-\beta_{0}\right)+\sqrt{n}\int \;h^{\text{*}}\left(y\right)d\left(\hat{A}-A_{0}\right)\left(y\right)\;=\sqrt{n}\left({\text{P}}_{n}-\text{P}\right)\text{S}\left(Y,Z\right)\left[v^{-},h^{-}\right]+o_{p}\left(\sqrt{n}\left\|\hat{\beta }-\beta_{0}\right\|+\sqrt{n}{\left\|\hat{A}-A_{0}\right\|}_{TV}\right)+o_{p}\left(1\right),$$

and this holds uniformly for $∥v^{*}∥\leq 1$ and ${∥h^{*}∥}_{TV}\leq 1$. Using (A9), we obtain that

$$\sqrt{n}\left\|\hat{\beta }-\beta_{0}\right\|+\sqrt{n}{\left\|\hat{A}-A_{0}\right\|}_{TV}=O_{p}\left(1\right).$$

Thus,

(A10)
$$\sqrt{n}v^{\text{*}T}\left(\hat{\beta }-\beta_{0}\right)+\sqrt{n}\int \;h^{\text{*}}\left(y\right)d\left(\hat{A}-A_{0}\right)\left(y\right)\;=\sqrt{n}\left({\text{P}}_{n}-\text{P}\right)\text{S}\left(Y,Z\right)\left[v^{-},h^{-}\right]+o_{p}\left(1\right).$$

This implies that

$$\sqrt{n}\left(\hat{\beta }-\beta_{0},\hat{A}-A_{0}\right),$$

as a random map on $\left(v^{*},h^{*}\right)$, converges weakly to a mean-zero and tight Gaussian process. Furthermore, by letting $v^{*}=1$ and $h^{*}=0$, we conclude that $\sqrt{n}\left(\hat{\beta }-\beta_{0}\right)$ has an influence function given by $S(Y,Z)\left[v^{-},h^{-}\right]$. Since the latter lies on the score space, it must be the efficient influence function. Hence, the asymptotic variance of $\sqrt{n}\left(\hat{\beta }-\beta_{0}\right)$ achieves the semiparametric efficiency bound. □
