# Supplementary material for: Asymptotic Properties for Cumulative Probability Models for Continuous Outcomes
Source: Mathematics (Basel). Author manuscript; Available in PMC 2024 Feb 19. (PMC10875740; doi:10.3390/math11244896)
Supplement: Supplement [file NIHMS1965607-supplement-Supplement.pdf]

Supplementary Material for Asymptotic Properties for Cumulative Probability Models for Continuous Outcomes

Chun Li[1], Yuqi Tian[2], Donglin Zeng[3], Bryan E. Shepherd[2]

[1]Division of Biostatistics, Department of Population and Public Health Sciences, University of Southern California
[2]Department of Biostatistics, Vanderbilt University
[3]Department of Biostatistics, University of Michigan
[1]Corresponding author (cli77199@usc.edu)

November 25, 2023

Table S1: Simulation results for estimates from CPMs on original data and on data categorized outside (L, U); $n = 5000$; based on 1,000 replicates

n	Estimand		Original Data	Data categorized outside (L, U) (e^{-4}, e^{4})	(e^{-2}, e^{2})	$(e^{-1/2}, e^{1/2})$
5000	β_1	bias	0.0014	0.0014	0.0012	0.0017
		SD	0.0302	0.0302	0.0304	0.0352
		mean SE	0.0300	0.0300	0.0302	0.0345
		MSE	0.0009	0.0009	0.0009	0.0012
	β_2	bias	-0.0006	-0.0006	-0.0004	-0.0009
		SD	0.0154	0.0154	0.0157	0.0189
		mean SE	0.0150	0.0150	0.0153	0.0181
		MSE	0.0002	0.0002	0.0002	0.0004
	$A(e^{0.5})$	bias	0.0019	0.0019	0.0018	0.0019
		SD	0.0248	0.0248	0.0249	0.0260
		mean SE	0.0242	0.0242	0.0243	0.0253
		MSE	0.0006	0.0006	0.0006	0.0007
	median$(Y \| X_1 = 0, X_2 = 0)$	bias	-0.0005	-0.0005	-0.0004	-0.0005
		SD	0.0245	0.0245	0.0246	0.0255
		MSE	0.0006	0.0006	0.0006	0.0007
	$E(Y \| X_1 = 0, X_2 = 0)$	bias	-0.0016	-	-	-
		SD	0.0372	-	-	-
		mean SE	0.0370	-	-	-
		MSE	0.0014	-	-	-

SD, standard deviation of replicates; mean SE, average estimated standard error across replicates; MSE, mean squared error.

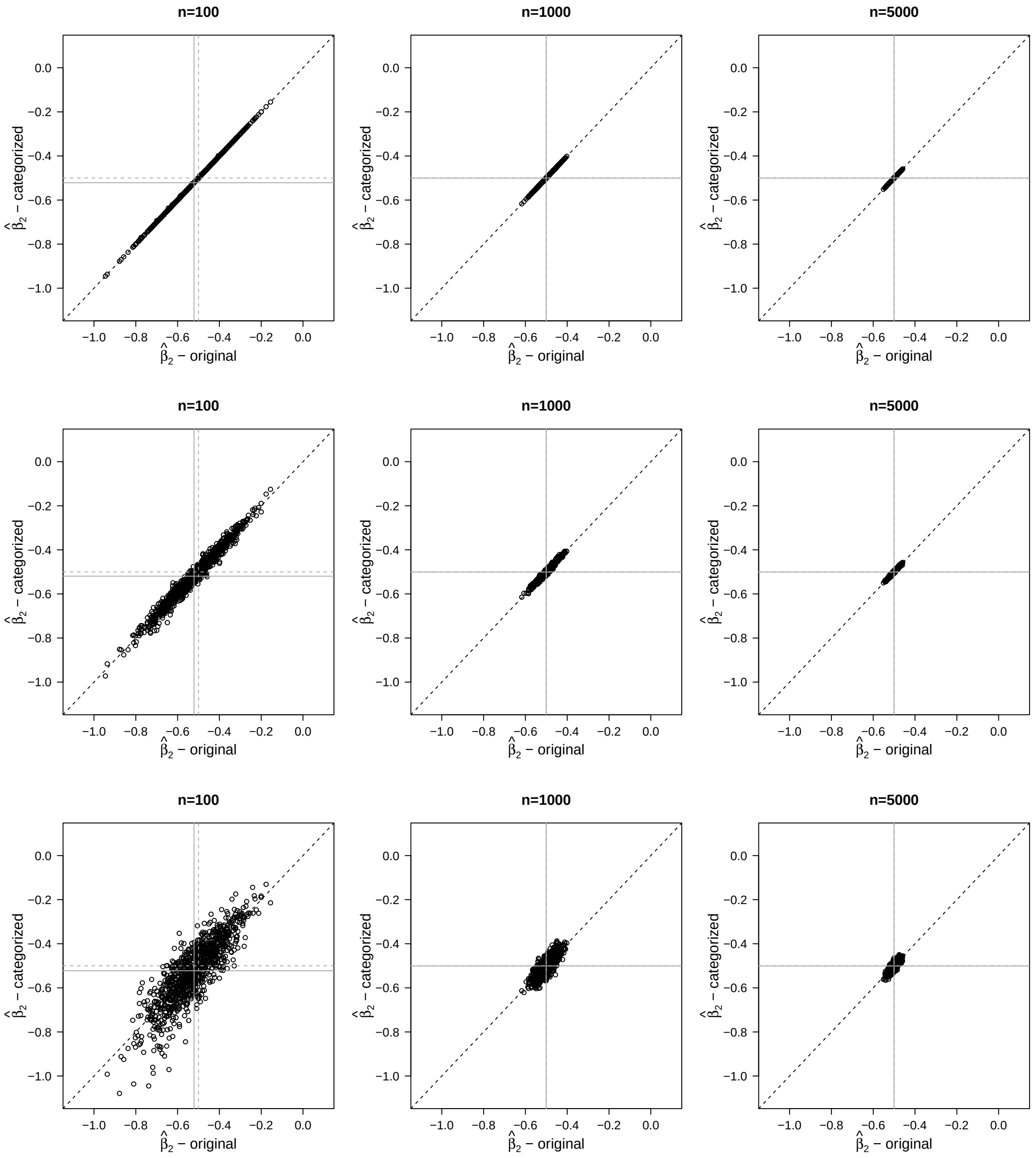

Figure S1: Estimates of β_2 using data categorized outside (L, U) compared to those using the original data and to the truth, $\beta_2 = -0.5$. Gray lines are mean estimates and dashed gray lines are the truth. Top row: $(L, U) = (e^{-4}, e^{4})$; middle row: $(L, U) = (e^{-2}, e^{2})$; bottom row: $(L, U) = (e^{-1/2}, e^{1/2})$. Left to right: $n = 100, 1000, 5000$. Based on 1000 replicates.

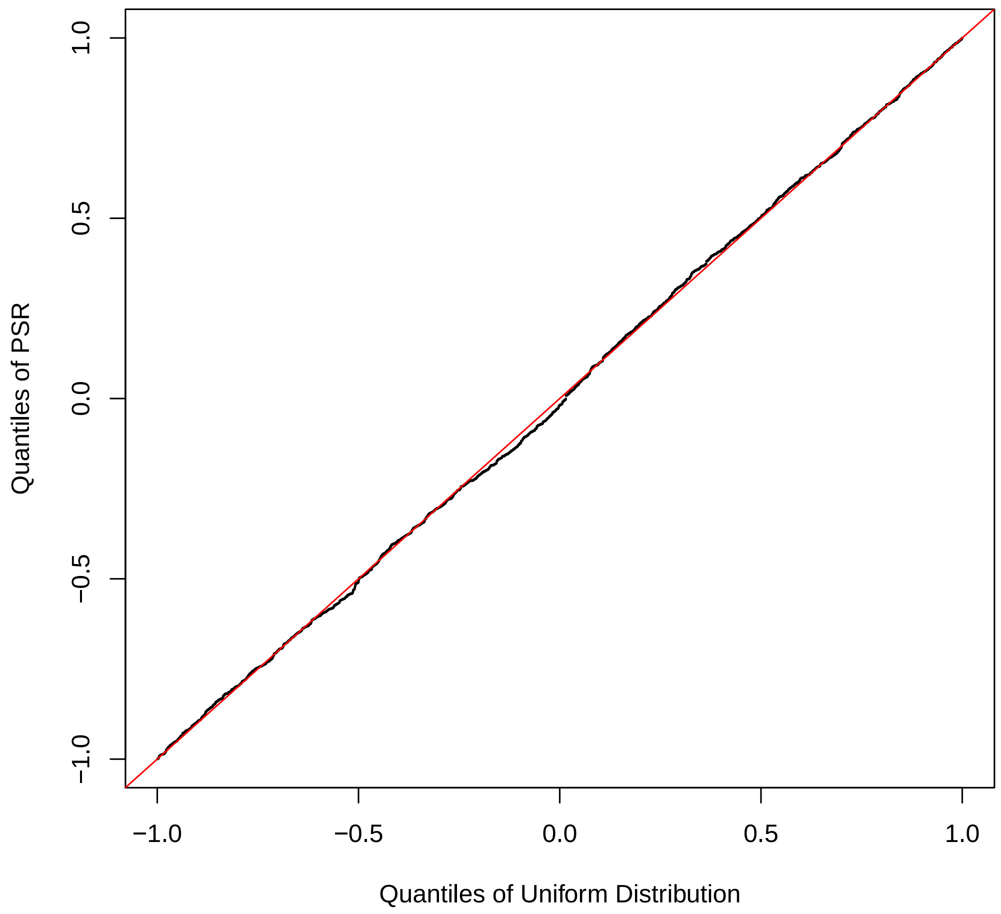

Figure S2: Quantile-quantile plot of probability-scale residuals (PSRs) from Model 3 for the example data suggests a good model fit.
